# Distinct accessory roles of *Arabidopsis* VEL proteins in Polycomb silencing

**DOI:** 10.1101/gad.350814.123

**Published:** 2023-09-01

**Authors:** Elsa Franco-Echevarría, Mathias Nielsen, Anna Schulten, Jitender Cheema, Tomos E. Morgan, Mariann Bienz, Caroline Dean

**Affiliations:** 1 Medical Research Council Laboratory of Molecular Biology, Cambridge CB2 0QH, United Kingdom;; 2 John Innes Centre, Norwich Research Park, Norwich NR4 7UH, United Kingdom

**Keywords:** VRN5, PRC2, Polycomb silencing, VIN3, VAL1, vernalization

## Abstract

Polycomb repressive complex 2 (PRC2) mediates epigenetic silencing of target genes in animals and plants. In *Arabidopsis*, PRC2 is required for the cold-induced epigenetic silencing of the *FLC* floral repressor locus to align flowering with spring. During this process, PRC2 relies on VEL accessory factors, including the constitutively expressed VRN5 and the cold-induced VIN3. The VEL proteins are physically associated with PRC2, but their individual functions remain unclear. Here, we show an intimate association between recombinant VRN5 and multiple components within a reconstituted PRC2, dependent on a compact conformation of VRN5 central domains. Key residues mediating this compact conformation are conserved among VRN5 orthologs across the plant kingdom. In contrast, VIN3 interacts with VAL1, a transcriptional repressor that binds directly to *FLC*. These associations differentially affect their role in H3K27me deposition: Both proteins are required for H3K27me3, but only VRN5 is necessary for H3K27me2. Although originally defined as vernalization regulators, VIN3 and VRN5 coassociate with many targets in the *Arabidopsis* genome that are modified with H3K27me3. Our work therefore reveals the distinct accessory roles for VEL proteins in conferring cold-induced silencing on *FLC*, with broad relevance for PRC2 targets generally.

## Abstract (precis)

In this study, Franco-Echevarria et al. describe the domain interactions between VEL protein, VRN5, and the core subunits of the Polycomb repressive complex 2 (PRC2) in *Arabidopsis*. They also describe the interactions between VEL protein, VIN3, and the transcriptional repressor VAL1, altogether expanding our understanding of the individual functions of VEL accessory proteins and their role in PRC2 assembly on chromatin for transcriptionally repressive histone methylation.

Polycomb repressive complex 2 (PRC2) is pivotal for the transcriptional silencing of genes that control development and differentiation in animals and plants. Silencing consists of an epigenetic memory mechanism based on the deposition of trimethylated lysine 27 of histone H3 (H3K27me3) at Polycomb target genes (Cao et al. 2002; Czermin et al. 2002; Müller et al. 2002). PRC2 comprises four subunits, each deeply conserved throughout animals and plants (Supplemental Fig. S1A; Birve et al. 2001; Tie et al. 2001). These cooperate to deposit the H3K27me3 silencing hallmark at *cis*-regulatory regions of targets and to copy it to daughter strands during DNA replication in a “read–write” mechanism (Yu et al. 2019). PRC2 accessory proteins are also required for long-term epigenetic memory when cell division rates are high (Yu et al. 2019; Lövkvist et al. 2021; Menon et al. 2021). They have been found to modulate the methyltransferase activity of PRC2 and/or promote tethering of the complex at genomic loci (Hauri et al. 2016; Choi et al. 2017; Chen et al. 2018; Kasinath et al. 2018; Oksuz et al. 2018; Højfeldt et al. 2019; van Mierlo et al. 2019).

In *Arabidopsis* and other plants, some of the PRC2 subunit functions are encoded by multiple genes; for example, the SUZ12 function can be provided by VRN2, EMF2, or FIS2. These are expressed in different developmental contexts (Mozgova and Hennig 2015; Godwin and Farrona 2022) or function in response to environmental changes. For example, the VRN2–PRC2 complex functions in vernalization, the cold-induced epigenetic silencing of *FLOWERING LOCUS C* (*FLC*) (Michaels and Amasino 1999; Hepworth and Dean 2015; Xu and Chong 2018; Costa and Dean 2019; Sharma et al. 2020). In contrast to the deep conservation of the PRC2 subunits (Supplemental Fig. S1A), the accessory proteins vary between animals and plants, often without obvious counterparts in the other kingdom. The *Arabidopsis* PRC2 accessory proteins VIN3 and VRN5 (VEL proteins) were identified genetically in *Arabidopsis* as genes required for *FLC* silencing (Sung and Amasino 2004; Greb et al. 2007) and physically associate with PRC2 in cold-treated plants along with their close relative, VEL1 (De Lucia et al. 2008; Kim and Sung 2010).

The hallmarks of VEL proteins are an atypical plant homeodomain (PHD) zinc finger, a fibronectin type III (FN3) domain, and a C-terminal VEL domain (Fig. 1A; Sung and Amasino 2004; Wood et al. 2006; Greb et al. 2007). We previously discovered that the VEL domain engages in spontaneous head to tail polymerization to assemble biologically relevant, dynamic biomolecular condensates (Fiedler et al. 2022). We have also determined the crystal structure of the PHD finger of VIN3 (using the homolog from palm trees, as this was the only VIN3 ortholog that was suitable for crystallization) and have shown that the PHD finger is the core module of a compact tripartite PHD superdomain (referred to here as PHDsuper) without histone H3 tail binding activity (Franco-Echevarría et al. 2022). In *Arabidopsis* plants, VIN3 expression is cold-inducible while VRN5 is constitutively expressed, and both become enriched at the Polycomb nucleation region of *FLC* during prolonged cold exposure (Sung and Amasino 2004; Yang et al. 2017). Their association with the *FLC* nucleation region in the cold confers metastable silencing, with H3K27me3 spreading across the whole locus upon return to warm temperatures to confer long-term epigenetic stability (Yang et al. 2017).

**Figure 1.**
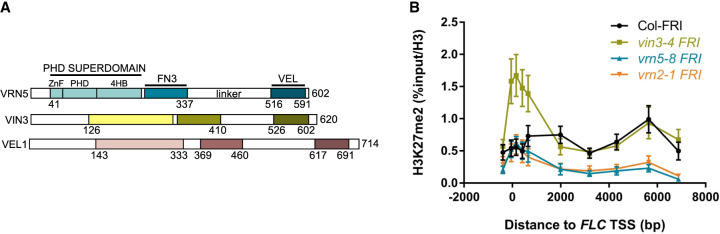
H3K27me2 enrichment at *FLC* in wt and mutant *Arabidopsis*. (*A*) Domain organization of VEL proteins. (*B*) Enrichment of H3K27me2 levels at *FLC* in Col*FRI* and different mutant plants vernalized for 6 wk (6WT0). Data are shown as the percentage input relative to H3. Nontransgenic Col*FRI* plants were used as a control sample. Error bars are means ± SEM from two independent experiments.

Recent studies have identified VEL relatives in other plants that do not undergo vernalization (Mohan et al. 2018; Yang et al. 2019; Fiedler et al. 2022; Shwartz et al. 2022), raising questions about the generality of VEL protein function and how they connect with PRC2 silencing. We therefore investigated the individual roles of the VEL proteins in PRC2 silencing, detailed VEL protein structural characteristics that underpin specific functionalities, and mapped the sites in the *Arabidopsis* genome where they are stably recruited. Our work revealed highly conserved features of different VEL paralogs that mediate differential interactions with PRC2 and other silencing proteins. The genome-wide colocalization of the VEL proteins at numerous other H3K27me3-enriched targets also suggests that VEL proteins have a broad role in PRC2 silencing during plant growth and development.

## Results

### Differential effects of *vrn5* and *vin3* on H3K27me2 dynamics

Methylation of H3K27 by PRC2 is intrinsically slow, so that H3K27me3 levels never reach their maximum before cell division occurs (Alabert et al. 2015; Berry et al. 2017). Therefore, the proposed roles of the PRC2 accessory proteins are to increase the catalytic activity of PRC2 toward its targets or to prolong its residency time at targets. Both of these roles are consistent with *vin3* and *vrn5* mutants accumulating no H3K27me3 at the *FLC* nucleation region in the cold. To explore the H3K27 methylation dynamics further, we investigated the intermediate methylation state, H3K27me2, at *FLC*. All experiments were undertaken in a FRIGIDA^+^ genotype, our standard vernalization-sensitive genotype (Berry et al. 2017), with different mutations or transgenes as specified. ChIP-qPCR showed no H3K27me2 enrichment in the *FLC* nucleation region in vernalized *vrn5* mutants (6WT0), with *vrn2* (defective in the PRC2 SUZ12 subunit) used as the benchmark control (Fig. 1B). Thus, VRN5 and VRN2 are strictly required for the PRC2-dependent deposition of H3K27me2 at *FLC*. Somewhat surprisingly though, *vin3* mutants showed accumulation of H3K27me2 in the nucleation region (Fig. 1B), revealing different roles for VIN3 and VRN5 in the PRC2-dependent silencing of *FLC*.

### VRN5 associates tightly with PRC2

**Figure 2.**
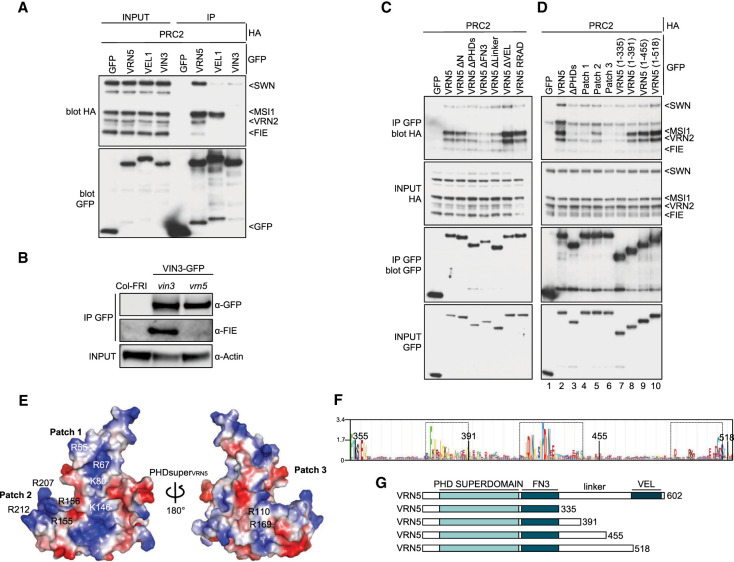
Analysis of the VRN5–PRC2 interaction. (*A*) Co-IP of HA-tagged PRC2 core components with GFP-tagged VEL proteins following coexpression in HEK293T cells, as indicated. (*B*) Western blots of α-GFP immunoprecipitates from extracts of vernalized *vrn5* mutant plants bearing a VIN3-GFP transgene, probed with α-FIE antibody. (*C*) Co-IP of HA-tagged PRC2 core components with wt or mutant GFP-VRN5 bearing internal domain deletions (ΔN, ΔPHDs, ΔFN3, Δlinker, and ΔVEL correspond to the deletions of the N-terminal region, PHDsuper, FN3 domain, flexible linker, and polymerizing VEL domain, respectively, as depicted in Fig. 1A) in HEK293T cells. (*D*) Co-IP of HA-tagged PRC2 core components with wt or GFP-VRN5 mutants in HEK293T cells as described in *G*. (*E*) Molecular surface representation of PHD_VRN5_ predicted by AlphaFold2, colored according to electrostatic potential ([red] negative [blue] positive), showing conserved positively charged residues forming clusters 1 and 2 (front surface) and cluster 3 (rear surface). (*F*) Sequence logo conservation analysis of VRN5 linker connecting the FN3 and VEL domains in Viridiplantae, with residue numbers shown. (Dashed squares) Three conserved regions (residues 371–391, 420–445, and 493–518 in *Arabidopsis*) are shown. (*G*) Schematic representation of different VRN5 linker constructs used in *D*.

Previous proteomic evidence indicated close association between VEL proteins and PRC2 (De Lucia et al. 2008; Qüesta et al. 2016), but whether these were direct was unknown. To address this, we coexpressed HA-tagged versions of all four PRC2 subunits (VRN2, SWN, FIE, and MSI1) (Supplemental Fig. S1B) with GFP-tagged VEL proteins in heterologous human embryonic kidney (HEK293T) cells (Fiedler et al. 2022) and monitored their associations by coimmunoprecipitation (co-IP). The results were reproducible across multiple experiments, with all four PRC2 subunits coimmunoprecipitating efficiently and robustly with GFP-VRN5 (Fig. 2A). Weaker and less robust co-IP was detected for PRC2 subunits with VIN3-GFP or GFP-VEL1 (except for a moderately strong co-IP of MSI1 with the latter) (Fig. 2A; Supplemental Fig. S1). This identifies VRN5 as the main and likely direct interaction partner of PRC2.

The lack of robust co-IP between VIN3 and PRC2 was surprising, since previous co-IP assays in plants revealed that VIN3 copurified with VRN2 in extracts from vernalized plants and cofractionated with PRC2 subunits during size exclusion chromatography (Wood et al. 2006; De Lucia et al. 2008). However, these apparent associations might have been bridged by endogenous VRN5. To test this, we probed VIN3-GFP immunoprecipitates from extracts of vernalized *vrn5* mutant plants for the presence of the PRC2 subunit FIE. We thus confirmed that FIE copurifies with VIN3 in a *vin3* mutant control line (pVIN3:VIN3-GFP in *vin3*) but fails to do so in a *vrn5* mutant line (Fig. 2B; Supplemental Fig. S1C). This is consistent with our co-IP results from HEK293T cells (Fig. 2A). It further suggests that the above-mentioned associations between VIN3 and PRC2 are likely mediated by VRN5, possibly by direct copolymerization between the VEL domains of VIN3 and VRN5 (Greb et al. 2007; Fiedler et al. 2022).

To further define the binding of VRN5 to PRC2, we tested individual GFP-VRN5 deletion mutants for their ability to coimmunoprecipitate PRC2. We thus found that PHDsuper, FN3, and the flexible linker between these domains enhance PRC2 association, whereas neither the VEL domain nor its ability to polymerize are required (Fig. 2C; Supplemental Fig. S1D; Fiedler et al. 2022). PHDsuper exhibits three surface patches formed by clusters of arginines (Rs) and lysines (Ks) that are not seen in canonical PHD fingers (Franco-Echevarría et al. 2022), which bind to positively charged histone H3 tails (Musselman and Kutateladze 2011; Sanchez and Zhou 2011). Substitutions of these positively charged R and K residues with glutamic acid (E) reduced (in the case of patch 2) or almost abolished (in the case of patch 1 or 3) PRC2 co-IP with GFP-VRN5, whereby the effects of patch 1 or 3 substitutions were as strong as the effect of deleting PHDsuper (Fig. 2D, lanes 1–6). Therefore, these positively charged surface patches, predicted to be in the front and back faces of PHDsuper by AlphaFold2 (Fig. 2E; Jumper et al. 2021), enhance the binding of VRN5 to PRC2. Crucially, the equivalent mutants of palm tree VIN3 PHDsuper are soluble, stable, and folded correctly, which indicates that the effects of these mutants in reducing PRC2 association reflect specific interaction defects. Note that patches 1 and 2 are near invariant among plant VEL proteins (Supplemental Figs. S2, S3; Franco-Echevarría et al. 2022), arguing for a widespread physiological relevance of these unusual positive surface patches of VEL PHD superdomains.

The requirement of the linker between VRN5 FN3 and VEL for the VRN5–PRC2 association was somewhat unexpected, as this linker (comprising 180 residues) is poorly conserved among VEL proteins (Supplemental Figs. S2, S3) and is predicted to be intrinsically disordered. A search for conserved residues within this linker using HMMER (Potter et al. 2018) identified three short conserved motifs (spanning residues 371–391, 420–445, and 493–518, respectively) within *Arabidopsis* VRN5 (Fig. 2F). To address whether any of these influence the VRN5–PRC2 interaction, we tested progressive internal deletions retaining increasing linker length (Fig. 2G). The efficiency of PRC2 association with GFP-VRN5 increased with progressively longer linker sequences (Fig. 2D, lanes 7–10). This suggests that this linker assists VRN5 in its binding to PRC2; e.g., by wrapping around the PRC2 complex. We also found that the N terminus of VRN5 (predicted to be structurally disordered) contributes to its association with SWN, MSI1, and VRN2, while its C-terminal 11 residues contribute to its association with SWN (Supplemental Fig. S1E), indicating that these additional flanking regions assist VRN5 in its binding to PRC2. The VRN5–PRC2 interaction is thus complex, involving the contribution of multiple domains and motifs within VRN5.

Finally, we also confirmed that the plant PRC2 complex, like its human counterpart (Schmitges et al. 2011; Kasinath et al. 2018), is capable of self-assembly, given that SWN, MSI1, and FIE associate with the GFP-tagged VRN2 (SUZ12) scaffold. Interestingly, if VRN5 is coexpressed with these PRC2 subunits, co-IP of MSI1 with GFP-VRN2 increases in a VRN5-dependent manner (Supplemental Fig. S1F,G), reinforcing the view that MSI1 contributes to at least one of the PRC2 docking points for VRN5.

### Different VEL orthologs are evolutionarily conserved throughout the plant kingdom

We have previously shown that PHDsuper and VEL domains are highly conserved throughout the plant kingdom (Fiedler et al. 2022; Franco-Echevarría et al. 2022). Exploring the conservation of the full-length VEL sequences revealed a high degree of conservation across all plant VEL paralogs. However, VRN5 orthologs differ the most from other VEL paralogs, as they are distinguishable from the latter by an invariant DLNxxxVPDLN motif in their linker (Supplemental Fig. S2, green dashed square). In contrast, there is a high degree of similarity between VIN3 and VEL1 orthologs, which together appear to constitute a subclass of VEL proteins (Supplemental Fig. S3). Indeed, VIN3 and VEL1 orthologs can only be differentiated from each other in Brassicaceae but cannot be differentiated in early diverging angiosperm species even if these contain multiple VIN3/VEL1-like genes (Supplemental Fig. S3). Notably, VRN5 and VIN3/VEL1 orthologs are present in both dicots and monocots, including plant species such as palm trees (*Phoenix dactylifera* [Pd]) or maize (*Zea mays* [Zm]) that do not require winter cold exposure for spring flowering. This suggests that the different VEL orthologs have widespread roles beyond vernalization in PRC2-dependent silencing.

### Understanding the binding preference of PRC2 for VRN5

**Figure 3.**
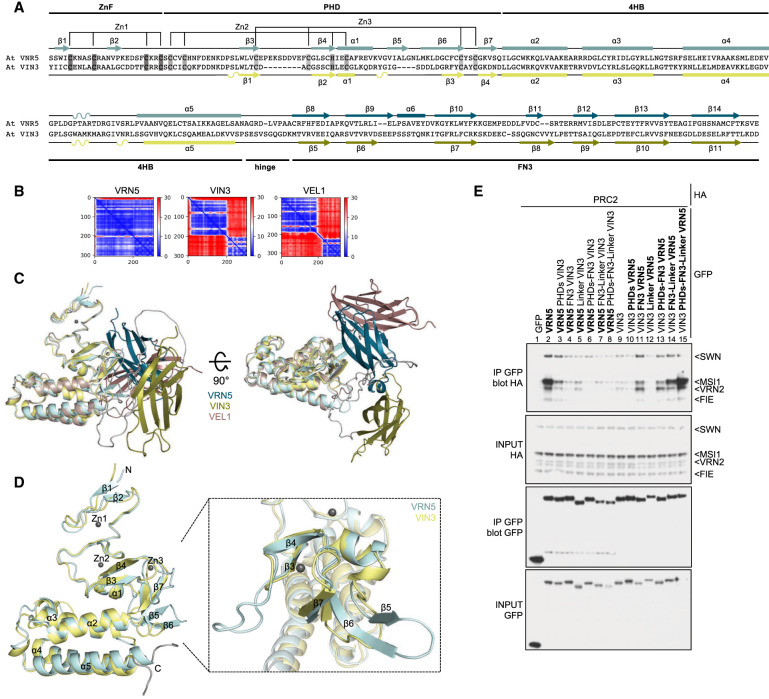
Structural differences between VRN5 and VIN3/VEL1 paralogs. (*A*) Sequence alignment between *Arabidopsis thaliana* (At) VRN5_41–339_ and At VIN3_126–412_, with predicted secondary structure indicated for VRN5 (*above*) and VIN3 (*below*). Highlighted are the Zn2^+^-ligating residues of ZnF and PHD finger. (*B*) PAE plot obtained for PHDsuper and FN3 domains of At VRN5_41–339_, At VIN3_126–412_, and At VEL1_143–462_. (Blue) Low error, (red) high error. See also Supplemental Fig. S4. (*C*) Orthogonal views of superpositions of At VRN5_41–339_ (blue), At VIN3_126–412_ (yellow), and At VEL1_143–462_ (brown) in ribbon representation, as predicted by AlphaFold2. The zinc ions are from Pd PHD_VIN3_ (PDB: 7QCE). (*D*) Superpositions of PHD superdomains of At VRN5_41–240_ (light blue) and VIN3_123–307_ (light yellow), as in *C*, with secondary structure elements indicated (Gray balls) Zinc ions (RMSD 1.70). Close-up view shows superimposed proximal PHD fingers as in *D*. (*E*) Co-IP of HA-tagged PRC2 core components with wt or mutant GFP-VRN5 or GFP-VIN3 bearing internal deletions or domain swaps (indicated at the *top*), as in Figure 2A.

The striking preference of PRC2 for VRN5 points to a specialized function of VRN5 orthologs. To understand why VRN5 is the only VEL protein capable of interacting with all four core PRC2 subunits, we used AlphaFold2 (Jumper et al. 2021) to predict the structures of different VEL paralogs in *Arabidopsis*. Interestingly, this revealed a major structural difference between VRN5 and VIN3 or VEL1; AlphaFold2 (https://alphafold.ebi.ac.uk) suggests a close packing of PHDsuper against FN3 exclusive to VRN5 orthologs, based on sequence conservation (Fig. 3A,B; Supplemental Figs. S2, S3). Indeed, VRN5 orthologs are predicted to adopt a compact conformation in a wide range of plants, whereas the conformation of VIN3 and VEL1 orthologs is predicted to be more open (Supplemental Fig. S4A). Furthermore, the predicted folds for various PHDsuper–FN3 modules closely resemble each other within the VRN5 lineage (Supplemental Figs. S4, S5A–C), suggesting that the interface between PHDsuper and FN3 is deeply conserved among all angiosperm VRN5 orthologs.

Examining the predicted PHDsuper folds of different VEL paralogs, we also found a major difference between VRN5 and VIN3 orthologs: VRN5 exhibits a bulky β-strand extension in the proximal region of the PHD finger, which is missing in VIN3 and VEL1 (Fig. 3A; Supplemental Figs. S2, S3). This extension is formed by VRN5 β6 (the topological equivalent of the shorter VIN3/VEL1 β3), which interacts with two shorter adjacent β-strands, β7 and β5, in an antiparallel fashion (whereby β5 is unique to VRN5 orthologs; Fig. 3C,D). Indeed, the known crystal structure of the PHDsuper from palm trees lacks this β-strand extension (Franco-Echevarría et al. 2022), as do the predicted PHD superdomains from other VIN3 or VEL1 orthologs (Supplemental Fig. S5D–F). This distinctive feature characteristic of VRN5 orthologs points to a pivotal role of this structural element in the intramolecular interaction with FN3 (see below).

To test the functional relevance of individual domains for the PRC2–VRN5 interaction, we generated GFP-tagged VRN5 and VIN3 chimeras in which individual domains were substituted with equivalent domains from the opposite type to monitor their ability to coimmunoprecipitate PRC2. We thus found that GFP-VRN5 chimeras bearing VIN3 domains were highly inefficient in coimmunopreciptating HA-PRC2 (Fig. 3E, lanes 1–8), while the gradual substitutions of domains in GFP-VIN3 with the corresponding VRN5 domains restored PRC2 co-IP in these assays, as expected (Fig. 3E, lanes 9–15). This is particularly striking in the case of the FN3 substitution in GFP-VIN3 (Fig. 3E, lane 11), which suggests that FN3 may confer the VRN5-specific interaction with PRC2 (see also below). In addition, the linker of VRN5 also contributes to its association with MSI1 (Fig. 3E, lane 14) and, together with PHDsuper and FN3, imparts efficient association with the whole PRC2 (and particularly with its VRN2 scaffold) (Fig. 3E, lane 2 vs. 15). These results suggest that VRN5 not only binds to the MSI1 subunit of PRC2 but also uses its PHDsuper–FN3 module to contact its VRN2 scaffold, whereby the latter may determine the binding preference of PRC2 for VRN5 (see below).

Finally, we note that the VRN2–PRC2 complex lacks the C2 domain present in SUZ12, the human counterpart of VRN2 (Gendall et al. 2001). Interestingly, this C2 domain contributes to the binding site of the mammalian PRC2 complex for its accessory factors PHF19 and AEBP2 (Chen et al. 2018, 2020). Strikingly, FN3 is a close structural relative of C2 (Supplemental Fig. S5G,H), suggesting that the plant FN3 domain in VRN5 mimics the C2 domain in the core PRC2 subunit in the human complex. Based on the results from our mutational analysis (Fig. 3E) and bearing in mind the structure of the human PRC2 complex (Kasinath et al. 2018), we propose that the VRN5 FN3 domain interacts mainly with the regulatory N-lobe of the VRN2 scaffold and with the MSI1 subunit of the plant PRC2 complex (see below). Further structural studies are required to fully elucidate these functional parallels.

### Intramolecular interactions between VRN5 PHDsuper and FN3

Our attempts to determine the crystal structure of the VRN5 PHDsuper–FN3 module from *Arabidopsis* and other plant species (including palm trees and maize) following expression in *Escherichia coli* were unsuccessful. We therefore resorted to cross-linking mass spectrometry (XL-MS) analysis to test whether we could detect an interaction between PHDsuper and FN3. We purified a PHDsuper–FN3 fragment from ZmVRN5 (because of the relatively high yields of the maize protein) and prepared the purified protein for XL-MS. This revealed multiple intramolecular cross-links between FN3 and the hinge region within FN3 itself and within the 4HB module of PHDsuper (Supplemental Fig. S6A–E). Overall, our XL-MS results support the notion of a substantial structural change following mutational disruption of the predicted interface between PHDsuper and FN3.

**Figure 4.**
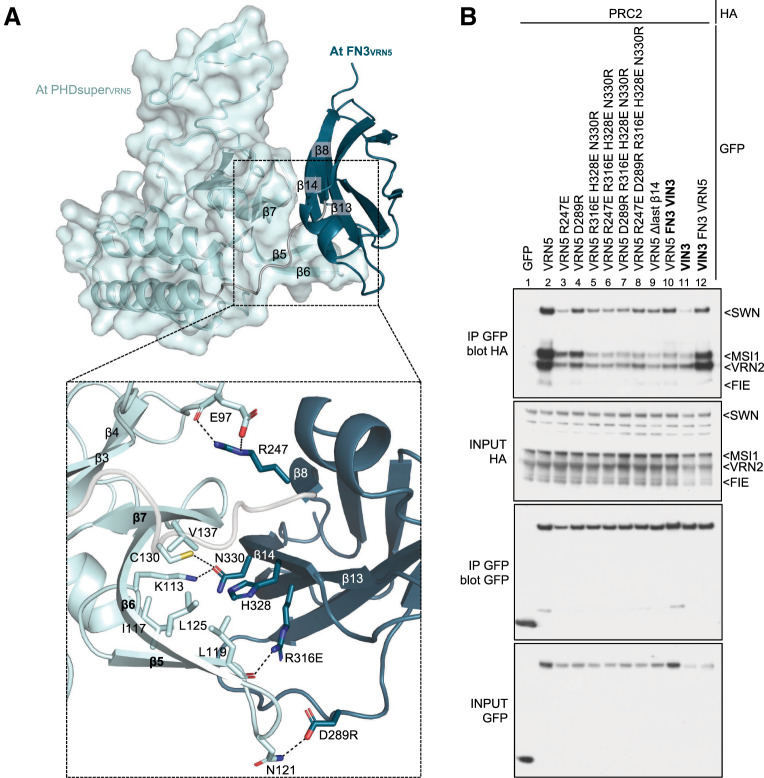
Analysis of intramolecular interdomain interactions within VRN5. (*A*) Structural prediction of At PHDsuper–FN3_VRN5_ (in ribbon and surface representations), with key structural elements mediating interdomain interactions highlighted. (Light blue) PHDsuper, (teal) FN3 domain. Close-up view of PHDsuper–FN3 interactions, with key interacting residues in stick representation. (Dashed lines) Hydrogen bonds or salt bridges. (*B*) Co-IP of HA-tagged PRC2 core components with wt or selected interdomain mutants of GFP-VRN5 (indicated at the *top*), as in Figure 2A.

The deep conservation of the four key residues within VRN5 FN3 (designated key quartet) that mediate its intramolecular interaction with PHDsuper (Fig. 4A; Supplemental Figs. S2, S6) indicates that the resulting compact conformation is inherent to all angiosperm VRN5 orthologs. To test the functional relevance of these FN3 residues, we introduced repelling point mutations into *Arabidopsis* GFP-VRN5 and tested the association of these mutants with PRC2 by co-IP (Fig. 4B). Remarkably, each of them strongly reduced PRC2 co-IP with GFP-VRN5 (Fig. 4B, lanes 2–8), similarly to deletion of the β14-strand of FN3 (Δβ14) (Fig. 4B, lane 9) that appears to engage in several close interactions with partner residues. Neither the key quartet nor β14 is conserved in VIN3 and VEL1 paralogs (Supplemental Fig. S3), consistent with our notion that the compact conformation conferred by these residues is unique to VRN5 orthologs. In summary, the compact conformation of *Arabidopsis* VRN5 described above is a defining structural feature of VRN5 orthologs that is functionally relevant for binding to PRC2.

### VRN5 binds predominantly to the regulatory lobe of PRC2

**Figure 5.**
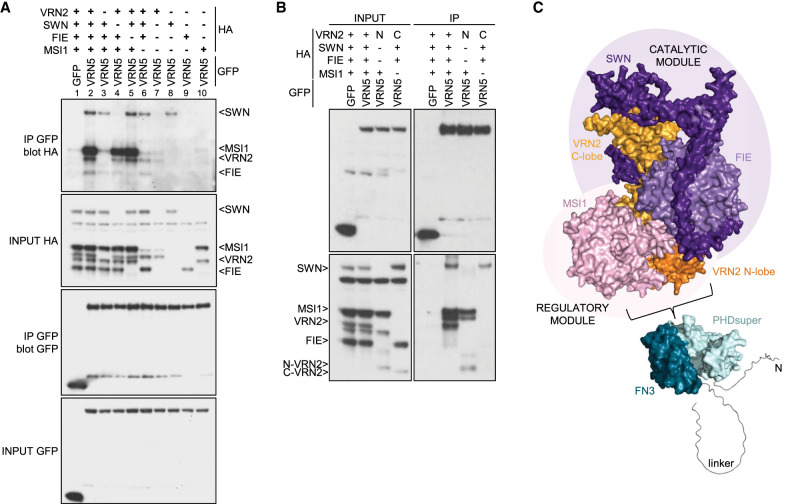
Interaction between VRN5 and the regulatory module of PRC2. (*A*) Co-IP of different combinations of HA-tagged PRC2 with GFP-VRN5, as in Figure 2A. (*B*) Co-IP of HA-tagged catalytic or regulatory modules of PRC2 with GFP-VRN5, as in Figure 2A. (N) VRN2 N-lobe, (C) VRN2 C-lobe. (*C*) Model of the interaction between structure prediction of VRN5 and the At PRC2 complex, with a catalytic module (purple background) comprising SWN (purple), FIE (light purple), and VRN2 C-lobe (light orange) and a regulatory module (light pink) comprising MSI1 (pink) and VRN2 N-lobe (orange).

The structure of the *Arabidopsis* PRC2 complex predicted by AlphaFold2 (Jumper et al. 2021) consists of a catalytic module comprising the histone methyltransferase SWN, FIE, and the C terminus of VRN2 (C-lobe) plus a regulatory module containing MSI1 and the N terminus of VRN2 (N-lobe), as seen in PRC2 complexes of other organisms (Chen et al. 2018; Kasinath et al. 2018; Grau et al. 2021). To establish the importance of each PRC2 subunit for their interaction with VRN5, we coexpressed GFP-VRN5 with all but one of the four PRC2 subunits for co-IP assays. This revealed that VRN2 and MSI1 strongly enhance the co-IP of PRC2 with GFP-VRN5 (Fig. 5A, lanes 3,6), whereas FIE does not (Fig. 5A, lane 5). Moreover, SWN is the only PRC2 subunit that associates with GFP-VRN5 independently of the others (Fig. 5A). Co-IP assays of GFP-VRN5 coexpressed with combinations of two individual PRC2 modules showed that VRN5 associates with the two components of the regulatory module; namely, MSI1 and the N-lobe of VRN2 (Fig. 5B). This suggests an extensive interface of VRN5 with the regulatory PRC2 module.

To test this notion, we performed XL-MS analysis of the purified VRN5ΔVEL–PRC2 complex expressed in SF9 insect cells (Supplemental Fig. S7). This revealed multiple cross-links between MSI1 and the N-terminal region of VRN5 that precedes its PHDsuper and also between the VRN5 4HB module and SWN and MSI1. Moreover, we found several cross-links between MSI1 and the upstream part of the unstructured linker region of VRN5 and between the latter and the N-terminal lobe of VRN2. Importantly, we found no cross-links between VRN5 and FIE, consistent with our co-IP data (Fig. 5A,B). Taken together, our XL-MS and co-IP results identified the regulatory module of PRC2 as the region of interaction for VRN5 (Fig. 5C), although further analysis will be required to determine the precise details of the molecular interaction between the two.

### VAL1 is dispensable for recruiting VIN3 to the FLC nucleation region

**Figure 6.**
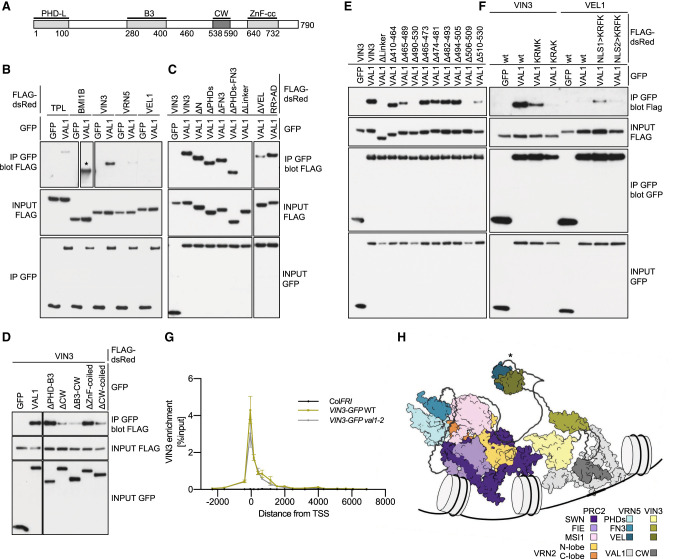
Association between VIN3 and VAL1. (*A*) Domain architecture of VAL1. (Dark gray) VIN3-interacting CW finger. (*B*–*F*) Co-IP of GFP-tagged wt or internal deletions of VAL1, with positive controls TPL and BMI1B tagged with FLAG-dsRed (star indicates short exposure) and wt or mutant FLAG-dsRed-tagged VEL proteins, revealing the critical role of VIN3_KRFK_ (Δ506–509) in *E*; note also that KRFK suffices to confer some VAL1 interaction on VEL1 in *F* (also see the text). ΔN, ΔPHDs, ΔFN3, Δlinker, and ΔVEL correspond to deletions of the N-terminal region, PHDsuper, FN3, flexible linker, and polymerizing VEL domain, respectively, as depicted in Figure 1A. RR>AD is a polymerization mutant (R554A R556D). (*G*) VIN3 enrichment at *FLC* in wt and *val1-2* mutant *Arabidopsis* vernalized for 6 wk (6WT0), with nontransgenic Col*FRI* as a negative control. Data are shown as percentage input; error bars are means ± SEM from two independent experiments. (*H*) Model based on AlphaFold2 predictions of the VRN5–PRC2 complex and its interaction with VIN3–VAL1 mediated by a heterotypic VEL–VEL interaction (star), with VAL1 being bound to the nucleation region of *FLC* (for simplicity, some flexible regions are not shown).

One boundary of the nucleation region, where VIN3 and VRN5 colocalize and where H3K27me3 accumulates, is the binding site for VAL1. VAL1 is a transcriptional repressor that acts as an assembly platform for cotranscriptional repressors and chromatin regulators (Veerappan et al. 2014; Qüesta et al. 2016; Yuan et al. 2016; Baile et al. 2021). VAL1 has a PHD-L domain, a DNA sequence-specific binding domain (B3), a CW domain, and a ZnF-EAR motif (Fig. 6A). Given the direct physical link between VAL1 and *FLC*, we asked whether we could detect a physical interaction between VAL1 and any of the VEL paralogs in co-IP assays. Indeed, monomeric dsRed-VIN3 showed robust co-IP with coexpressed GFP-VAL1, whereas dsRed-VRN5 and dsRed-VEL1 showed very little (Fig. 6B). This VIN3–VAL1 association depends on the linker region and CW domain of VAL1 (Fig. 6C,D), whereby the CW domain is a PHD-like zinc finger whose human homologs bind to H3K4me (Liu et al. 2016). It also depends on a short KRFK motif in VIN3 within the flexible linker separating FN3 and VEL, and additional flanking sequences in both proteins also contribute to a robust VIN3–VAL1 interaction (Fig. 6E; Supplemental Fig. S8A–E).

Intriguingly, the KRFK motif conforms to a classical monopartite nuclear localization signal (cNLS; K K/R X K/R) (Chelsky et al. 1989), which confers strong and specific binding between cNLS-containing cargo and importin (Conti and Kuriyan 2000; Hodel et al. 2001; Marfori et al. 2012). Consistent with this, deletion of KRFK renders dsRed-VIN3 cytoplasmic, but its nuclear localization and association with VAL1 can be restored by a heterologous viral NLS (PKKKRKV) inserted next to the N-terminal tag (Supplemental Figs. S8, S9), perhaps because of the sequence resemblance between the two motifs. Importantly, however, substitution of KRFK in dsRed-VIN3 with KRMK or KRAK strongly reduces or abolishes its co-IP with GFP-VAL1 (Fig. 6F), although both substitutions retain their nuclear localization (Supplemental Fig. S9), as expected since the third is the least important residue in cNLS motifs in regard to their affinities to importin (Hodel et al. 2001; Marfori et al. 2012). In contrast, the third residue within KRFK is clearly a critical determinant for the association of VIN3 with VAL1.

VEL1 contains two putative cNLS motifs—one upstream of its PHD finger (KRQR) and another downstream from it (KRMK) (Supplemental Fig. S8F)—yet poorly coimmunoprecipitates with GFP-VAL1 (Fig. 6B). Again, this argues against the notion that the nuclear localization of VIN3 per se enables it to bind to VAL1. In further support of this, dsRed-VEL1 mutants bearing a KRFK substitution of either motif alone does not affect their nuclear localization (Supplemental Fig. S9), but the upstream—albeit not the downstream—KRFK substitution confers weak yet readily detectable co-IP with GFP-VAL1 (Fig. 6F). Evidently, KRFK is sufficient to confer modest VAL1 association on VEL1 in a context-sensitive manner (note that the downstream substitution may be too close to FN3 to allow it to interact with VAL1) (Supplemental Fig. S8F).

VIN3-GFP is associated with the nucleation region in control plants vernalized for 6 wk (6WT0) (Yang et al. 2017). We therefore tested whether this was reduced in *val1-2*. Interestingly, loss of VAL1 did not change VIN3 association at the nucleation region (Fig. 6G). Thus, in vivo association of these two proteins may be highly dynamic or part of a multifactorial assembly complex, where other factors, potentially including VAL paralogs, can substitute for loss of VAL1. The observation that VIN3 is dispensable for the deposition of H3K27me2 at *FLC* (Fig. 1B) and that VAL1 is dispensable for VIN3 tethering at *FLC* (Fig. 6G) implies that the methyl transferase activity of VRN2–PRC2 can be targeted to the *FLC* nucleation region in the absence of VAL1 or VIN3. In support of this notion, a mutation preventing binding of VAL1 (C585T) (Qüesta et al. 2016) still accumulates H3K27me2 at the *FLC* nucleation region (Supplemental Fig. S10). Presumably, this targeting event is achieved by a combination of factors that associate directly with this key *cis*-regulatory region of *FLC*. Thus, this region can be likened to the Polycomb response elements in *Drosophila* that are *cis*-linked to PRC2 target genes, where multivalent interactions determine stable PRC association (Kassis and Brown 2013).

Given that VIN3-dependent polymerization is required for the silencing of *FLC* in the cold (Fiedler et al. 2022) and our new results suggesting that VIN3 facilitates the PRC2-mediated conversion of H3K27me2 to H3K27me3, a potential model is that VIN3 facilitates stable tethering of VRN5–PRC2 to *FLC* to facilitate the slow, gradual conversion of H3K27me2 to H3K27me3, possibly through VEL-dependent copolymerization with VRN5 (Fig. 6H).

### VEL proteins colocalize at numerous sites throughout the *Arabidopsis* genome

**Figure 7.**
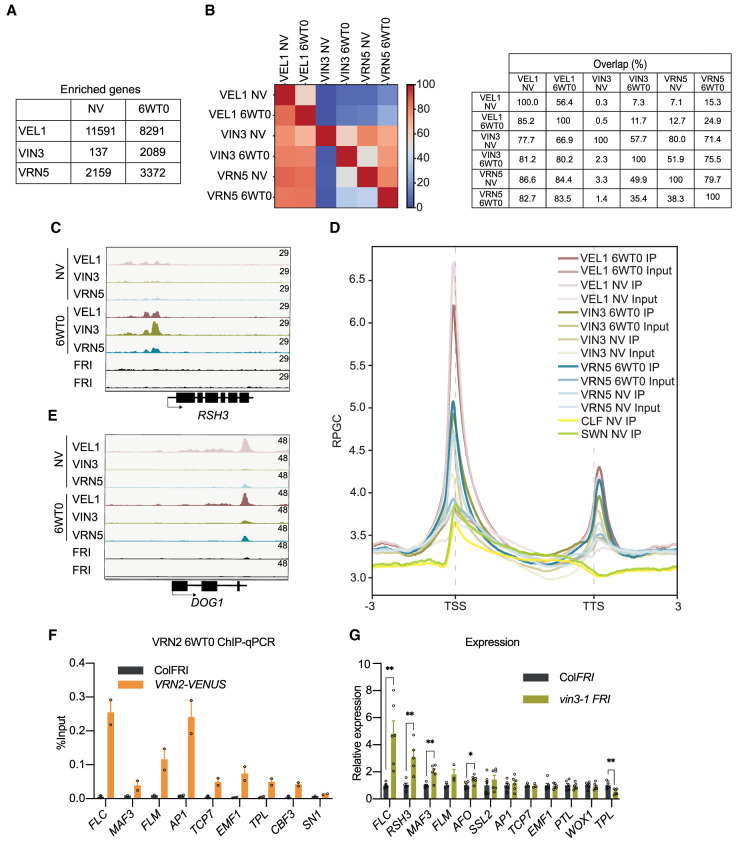
Genome-wide occupancy of VEL1, VIN3, and VRN5. (*A*) Table showing the number of VEL1, VIN3, and VRN5 target genes at NVs and 6WT0. Target genes present in at least two replicates were counted. We called 47,547 and 30,492 VEL1 peaks and assigned them to 11,591 and 8291 genes at NVs and 6WT0, respectively. We identified 175 peaks/137 genes (NVs) and 5672 peaks/2089 genes (6WT0) as potential targets for VIN3, and 6185 peaks/2159 genes (NVs) and 12,133 peaks/3372 genes (6WT0) as potential targets for VRN5. (*B*) Heat map showing the multiple overlaps among the VEL1, VIN3, and VRN5 at NVs and 6WT0 peaks (*left*) and the percentage overlap between peaks identified for VEL1, VIN3, and VRN5 at NVs and 6WT0 (*right*). (*C*) IGV screenshots showing the colocalization of the VEL proteins at the TSS of *RSH3*. (*D*) Metagene plots of VEL distribution over transcription units and flanking regions. (TSS) Transcription start site, (TTS) transcription termination site, (RPGC) reads per genomic content. Inputs from the respective VEL IPs were used as controls. (*E*) IGV screenshots showing the colocalization of the VEL proteins at the TTS of *DOG1*. (*F*) ChIP-qPCR showing enrichment of VRN2 after 6 wk of cold treatment (6WT0) at 10 potential target genes. Data are shown as the percentage input. Nontransgenic Col*FRI* plants were used as a negative control sample. *FLC* was used as a positive control locus, and *SN1* was used as a negative control locus. Error bars are means ± SEM from two independent experiments. (*G*) Expression of several potential target genes in the mutant *vin3-1* FRI relative to the wt Col*FRI* at 6WT0. Data are shown normalized to the expression level of the respective gene in Col*FRI*. Error bars are means ± SEM from at least three biological replicates. (*) *P* < 0.05, (**) *P* < 0.01 for statistical tests between samples by Student's *t*-test.

To pursue the generality of VEL protein function and how the proteins connect with PRC2 silencing, we undertook ChIP-seq analysis using lines expressing VIN3-GFP, VRN5-YFP, or VEL1-3xFLAG in either nonvernalized plants (NVs) or after 6 wk of cold (6WT0). We obtained at least 30 million total reads per sample with mapping rates of 55%–92% (Supplemental Data Set S1). After peak calling with MACS3, we only considered highly significant peaks (*Q*-value ≤10^−10^) for further analysis. A VEL peak located within 1 kb upstream of the TSS to 1 kb downstream from the TTS was assigned to that gene. Particularly for VIN3 but also for VRN5, the number of target genes was much higher after 6 wk of cold in comparison with NVs (Fig. 7A). This may reflect increased VIN3 levels, thus increasing the residency time of not only VIN3 but also VRN5 at many target genes, including *FLC* (Supplemental Fig. S11A). We identified many more potential target genes for VEL1 than for VIN3 and VRN5 (Fig. 7A; Supplemental Data Set S2). The different tags (FLAG on VEL1 vs. GFP or YFP on VIN3 or VRN5, respectively) likely contributed to the different enrichments of these proteins. Also, VEL1 associates well with MSI1 (Fig. 2A) and functions in other MSI1 complexes as well as PRC2; e.g., MSI1–HDAC complexes (Ning et al. 2019; Chen et al. 2023). We performed an overlap analysis of binding peaks, which showed that a large majority of VIN3 and VRN5 peaks were co-occupied by VEL1 both before and after cold treatment (e.g., at 6WT0, 80.2% of VIN3 peaks and 83.5% of VRN5 peaks colocalized with VEL1 peaks) (Fig. 7B).

An additional 1497 genes have recently been reported to have increased H3K27me3 levels after cold exposure (Xi et al. 2020). One example is *RSH3* (At1G54130), which has been linked to down-regulation of chloroplast transcription in response to abscisic acid (Yamburenko et al. 2015). Like *FLC*, *RSH3* shows an obvious increase in ChIP signal of VIN3 but also of VEL1 and VRN5 after cold treatment (Fig. 7C). We reanalyzed the data from Xi et al. (2020)—the only H3K27me3 data set generated in a FRIGIDA^+^ genotype to date—with the same peak calling pipeline as used in our analysis with VEL proteins. We were able to identify only 635 loci specifically enriched for H3K27me3 after vernalization and so did not continue the analysis.

To further evaluate the degree to which VEL proteins are linked to PRC2 function, we analyzed the sites of VEL protein enrichment at genes. This revealed that VEL proteins preferentially enrich at transcription start sites (TSSs) (Fig. 7D), with a smaller but significant peak frequently detected slightly downstream from the transcription termination site (TTS) (Fig. 7D). For example, at *DELAY OF GERMINATION 1* (*DOG1*), there is strong enrichment of VEL1 and, to some extent, of VIN3 and VRN5 at the TTS (Fig. 7E). We then reanalyzed ChIP-seq data obtained for the core PRC2 proteins SWN and CLF (Shu et al. 2019) with our analysis pipeline. This confirmed that CLF and SWN are mostly found at the TSS like the VEL proteins (Fig. 7D).

Comparison with other genome-wide *Arabidopsis* H3K27me3 data sets comes with the caveat that they used the Col*fri* genotype mutant for FRIGIDA, and FRIGIDA antagonizes H3K27me3 accumulation at *FLC* (Yang et al. 2016). We thus limited comparison to 1733 potential VEL target genes common between VEL1 and VRN5, but not VIN3, in NV conditions. This revealed an overlap of ∼18% between H3K27me3 enrichment and genes bound by VEL1 and VRN5 (Supplemental Fig. S11B; Zhang et al. 2007; Li et al. 2015; Zhou et al. 2017; Shu et al. 2019). A large number of these target genes also overlap with the PRC1-mediated modification H2AK121ub (Supplemental Fig. S11C; Zhou et al. 2017). Comparison of VEL1-only targets in nonvernalized seedlings showed a significant overlap with genes covered by H2AK121ub, with 16% showing both H2AK121ub and H3K27me3, reinforcing an important function for VEL1 in Polycomb function (Supplemental Fig. 11D).

The functional significance of the VEL ChIP-seq data is best elaborated at the well-established PRC2 target *FLC* and its *MAF1–MAF5* relatives (Ratcliffe et al. 2001, 2003). At *FLC*, VEL1, VIN3, and VRN5 are found in the nucleation region, as expected for a 6WT0 treatment (Supplemental Fig. S11A; Yang et al. 2017). The enrichment of VEL1 at *FLC* resembles the pattern for VRN5: enriched at the nucleation region in NV conditions and becoming more enriched with cold exposure (Supplemental Fig. S11E). *MAF1/FLM*, *MAF2*, and *MAF3* are repressed by vernalization, while *MAF4* and *MAF5* are not (Sheldon et al. 2009; Kim and Sung 2013); *MAF5* is induced by vernalization (Ratcliffe et al. 2003). Consistent with this, we found that VIN3 was enriched at *FLM*, *MAF3*, and to some extent *MAF2* (Supplemental Fig. S11A) but not at *MAF4* and *MAF5*. VRN5 was enriched at *FLM* but not at *MAF2*–*MAF5*, consistent with *vrn5* mutants mostly affecting *FLC* expression and to some extent *FLM* but not other members of the *FLC* gene family (Kim and Sung 2013). Likewise, VEL1 is enriched at *MAF4* and *MAF5* in addition to *FLC* and to some extent *FLM* (Supplemental Fig. S11A), consistent with *MAF4* and *MAF5* being reported to be misregulated in *vel1* mutants (Kim and Sung 2013). We note that some enrichment is also found at the 3′ end of *MAF5*, which might reflect the different behavior of *MAF5* during vernalization compared with the other *FLC* clade members. These differential enrichments point to the varied importance of VEL proteins in PRC2 silencing at different loci.

We used ChIP-qPCR to validate an additional selection of the targets identified by ChIP-seq (Supplemental Fig. S11F). In addition, to link their regulation to PRC2, we also tested for colocalization of VRN2 using a transgenic line expressing Venus-tagged VRN2 (VRN2-Venus) that complements the *vrn2-1* mutant. VRN2-Venus enrichment was detected at all selected VEL targets (Fig. 7F). Genes showing strong enrichment of VIN3 were also frequently misregulated in a *vin3* mutant background (Fig. 7G) and to a lesser extent in the *vrn5* mutant (Supplemental Fig. S11G). Consistent with VIN3 working with PRC2, in four out of five cases, we observed a release of repression in the *vin3-1* mutant, similar to previous studies analysing misregulation in PRC2-defective mutants (Shu et al. 2019).

## Discussion

We discovered that different VEL paralogs (cold-induced VIN3, constitutively expressed VRN5, and VEL1) are evolutionarily conserved and have functionally distinct activities important for Polycomb silencing. VRN5 extensively interacts with multiple subunits of PRC2, likely directly, enabling H3K27me2 and H3K27me3 deposition at the nucleation region of *FLC*. VEL1 may contribute to this role. In contrast, VIN3 interacts with VAL1 as part of a multifactorial assembly platform coordinating various activities for epigenetic silencing of *FLC*, likely by connecting PRC1 and PRC2 activities (Yuan et al. 2021; Mikulski et al. 2022) and facilitating the PRC2-mediated conversion of H3K27me2 to H3K27me3. Homopolymerization and potentially heteropolymerization of VEL domains of all VEL paralogs could promote dynamic biomolecular condensation of Polycomb complexes (Fiedler et al. 2022), thereby imparting a high binding avidity on these complexes (Bienz 2014, 2020; Pancsa et al. 2019). Our model is that VEL proteins work collectively, contributing to PRC2 stabilization and chromatin association and enabling the transition from H3K27me2 to H3K27me3. Although this mechanism has been elaborated predominantly at the *FLC* locus, their multiple targets and evolutionary conservation suggest a broad role for VEL proteins in plant growth and development.

## Materials and methods

### Generation of plasmids

VEL sequences (VIN3, Q9FIE3; VEL1, Q9SUM4; and VRN5, Q9LHF5) for in vitro and cell-based assays were amplified by polymerase chain reaction (PCR) from either plasmid templates (Greb et al. 2007) or synthetic genes (gBlocks, IDT) and cloned into mammalian or bacterial expression vectors by restriction-free cloning. Point mutations and deletions were generated by QuikChange using KOD DNA polymerase (Merck Millipore). All plasmids were verified by sequencing.

### Protein expression and purification

PHDsuper–FN3_VRN5_ from Zm for XL-MS analysis was inserted into pEC-LIC-His-3C containing a hexahistidine tag and a 3C protease cleavage site at the N terminus and was expressed in BL21 CodonPlus (DE3)-RIL cells (Agilent) in LB medium. Cells were grown at 37°C to OD_600_ 0.6 and then moved to 18°C, followed by induction with 0.4 mM IPTG and 100 μM ZnCl_2_ at OD_600_ 0.8. Harvested cells were resuspended in lysis buffer (25 mM Tris at pH 8.0, 200 mM NaCl, 20 mM imidazole at pH 8, EDTA-free protease inhibitor cocktail [Roche]) and lysed by sonication (Branson). Cleared lysates were loaded onto Ni-NTA resin (Qiagen) and washed with lysis buffer. After extensive washing, samples were eluted with lysis buffer supplemented with 300 mM imidazole. Eluted samples were incubated with 2 mM DTT and cleaved by 3C protease (made in-house; protease:protein ratio 1:80) overnight at 4°C. After overnight incubation with 3C, the sample was loaded onto a HiLoad 26/600 Superdex 200-pg column (GE Healthcare) equilibrated in 25 mM Tris (pH 8), 150 mM NaCl, and 1 mM DTT. All the purifications process were done at 4°C.

Pd PHD_VIN3_ patch mutants (patch 1: residues R142E, R154E, K167E, and K233E; patch 2: residues R242E, R243E, R294E, and R299E; and patch 3: residues K197E, K256E, K265E, and K276E) were expressed and purified as described before (Franco-Echevarría et al. 2022).

A VRN5ΔVEL–PRC2 complex was coexpressed in SF9 insect cells using the biGBac method (Weissmann et al. 2016). N-terminal strep tag II-tagged VRN5ΔVEL (minimal VRN5 construct that showed the strongest PRC2 interaction in our co-IP experiments); N-terminal HA-tagged VRN2_1–397_, SWN_29–831_, and FIE_1–360_; and C-terminal HA-tagged MSI1_1–412_ were cloned separately into a pLIB vector. The five genes were subsequently subcloned into a pBIG1a vector by a Gibson assembly reaction in which these gene PCR products were connected in series with the linearized pBIG1a vector digested by SwaI. The recombinant baculovirus was generated using FuGENE HD transfection reagent (Promega) in Sf9 cells using insect X-press (Lonza) medium, and infected cells were grown for 60–72 h at 27°C before being harvested for protein extraction and purification. Harvested cells were resuspended in 25 mM HEPES (pH 8), 250 mM NaCl, 2 mM MgCl_2_, 1 mM DTT, 5% glycerol, and EDTA-free protease inhibitor cocktail (Roche) and lysed by sonication. After centrifugation at 15,000 rpm for 30 min, the supernatant was incubated with Strep-tactin superflow resin (Qiagen) for 2 h, extensively washed with lysis buffer, eluted in lysis buffer with 5 mM desthiobiotin, and further purified by size exclusion chromatography with a Superose 6 increase 10/300 GL column (GE Healthcare).

### Phylogenetic analysis

Protein sequences of VEL orthologs were obtained from BLAST (Camacho et al. 2009) or retrieved from JACKHMMER (https://www.ebi.ac.uk/Tools/hmmer/search/jackhmmer). Alignments of sequences were done with MacVector (MacVector, Inc.) and ESPRIPT using Clustal Omega (https://www.ebi.ac.uk/Tools/msa/clustalo).

### AlphaFold2 predictions

Structure predictions and PAE plots were calculated from ColabFold AlphaFold2 using MMseqs2 (https://colab.research.google.com/github/sokrypton/ColabFold/blob/main/AlphaFold2.ipynb).

### XL-MS

Protein cross-linking reactions were carried out for 60 min at room temperature with 50 mg of complex present in 1 mM, 5 mM, 10 mM, and 20 mM EDC. Cross-linked protein was quenched with the addition of Tris buffer to a final concentration of 50 mM. The quenched solution was reduced with 5 mM DTT and alkylated with 20 mM iodoacetamide. SP3 protocol as described in Batth et al. (2019) and Hughes et al. (2019) was used to clean up and buffer-exchange the reduced and alkylated protein, and proteins were washed with ethanol using magnetic beads for protein capture and binding. The proteins were resuspended in 100 mM NH_4_HCO_3_ and digested with trypsin (Promega) at an enzyme to substrate ratio of 1:25 and 0.1% mazimum protease (Promega). Digestion was carried out overnight at 37°C. Cleanup of peptide digests was carried out with HyperSep SpinTip P-20 (Thermo Scientific) C18 columns using 80% acetonitrile as the elution solvent. Peptides were then evaporated to dryness via speed vacuum. Samples were fractionated via size exclusion chromatography using a Superdex 30 Increase 3.2/300 column (GE Healthcare) at a flow rate of 20 µL of 30% ACN and 0.1% TFA per minute. Fractions were taken every 3 min; fractions 1–3, 4–6, and 13–15 were concatenated; fractions 7–12 were analyzed separately; and all samples were dried on a speed vacuum. Dried peptides were suspended in 3% acetonitrile and 0.1% formic acid and analyzed by nanoscale capillary LC-MS/MS using an Ultimate U3000 HPLC (Thermo Scientific) to deliver a flow of 300 nL/min. Peptides were trapped on a C18 Acclaim PepMap100 5-μm, 100-μm × 20-mm nanoViper (Thermo Scientific) before separation on a PepMap RSLC C18 2-μm, 100-A, 75-μm × 50-cm EasySpray column (Thermo Scientific). Peptides were eluted on a 110-min gradient with acetonitrile and interfaced via an EasySpray ionization source to a quadrupole Orbitrap mass spectrometer (Q-Exactive HFX, Thermo Scientific). MS data were acquired in data-dependent mode with a Top 10 method, and high-resolution full-mass scans were carried out (*R* = 120,000, *m/z* 400–1550) followed by higher-energy collision dissociation (HCD) with a stepped collision energy range of 26%, 30%, and 34% normalized collision energy. The tandem mass spectra were recorded (*R* = 60,000, AGC target = 1 × 105, maximum IT = 120 msec, isolation window *m/z* 1.6, and dynamic exclusion 50 sec). For cross-linking data analysis, Xcalibur raw files were converted to MGF files using ProteoWizard (Chambers et al. 2012), and cross-links were analyzed by MeroX (Götze et al. 2015). Searches were performed against a database containing known proteins within the complex to minimize analysis time with a decoy database based on peptide sequence shuffling/reversing. Search conditions used a maximum of three missed cleavages with a minimum peptide length of five amino acids; cross-linking targeted residues were K, S, T, and Y; and cross-linking modification masses were 54.01056 Da and 85.98264 Da. Variable modifications were carbmidomethylation of cysteine (57.02146 Da) and methionine oxidation (15.99491 Da). False discovery rate was set to 1%, and assigned cross-linked spectra were manually inspected.

### Co-IP assays in human cells

Protein coimmunoprecipitation assays were carried out in HEK293T cells grown on DMEM supplemented with 10% FBS and seeded on poly-L-lysine-coated plates at ∼70% confluency using 89-mm tissue culture dishes per co-IP. After attachment, cells were transfected with a DNA:PEI (1:3.5) mixture. With the purpose of achieving near-stoichiometric expression of each protein in PRC2-VRN5 co-IPs, 10 μg of total DNA was used per culture dish (2 μg of GFP-VRN5, 4.3 μg of VRN2, 2.4 μg of SWN, 0.3 μg of MSI1, and 1 μg of FIE). For VAL1 and VEL protein co-IPs, 8 μg of total DNA (2 μg of GFP-VAL1 and 6 μg of FLAG-dsRed-TPL/BMI1B [dsRed-monomer N1 vector] or VEL proteins) was used. Cells were lysed ∼18 h after transfection in lysis buffer (20 mM Tris at pH 7.4, 200 mM NaCl, 10% glycerol, 5 mM NaF, 2 mM Na_3_VO_4_, 1 mM EDTA, 0.2% Triton X-100, EDTA-free protease inhibitor cocktail [Roche]). Lysates were clarified by centrifugation at 16,100 rcf for 20 min, and supernatants were incubated with GFP-trap (Chromotek) for 90 min at 4°C on an overhead tumbler. Immunoprecipitates were washed three times in lysis buffer and eluted by boiling in LDS sample buffer for 10 min. Input and co-IP fractions were separated by SDS-PAGE, blotted onto PVDF membrane, checked for equal loading by Ponceau staining, and processed for Western blotting with the appropriate antibodies. Primary antibodies (anti-GFP [Sigma G1544], anti-HA [Abcam ab9110], and anti-FLAG [Sigma 7425]) and secondary antibodies were diluted 1:5000 in PBS, 0.1% Triton X-100, and 5% milk powder. Blots were washed with PBS and 0.1% Triton X-100 and developed with ECL Western blotting detection reagent on film.

### Immunofluorescence

HEK293T cells were transfected with 1 mg of total DNA and 3.5× PEI. HEK293T cells were transfected with 1 μg of total DNA (250 ng of GFP-VAL1 and 750 ng of FLAG-dsRed-VIN3 and FLAG-dsRed-VEL1 constructs) and expressed for 18 h. PBS-washed cells were fixed on coverslips with 4% formaldehyde in PBS for 20 min and subsequently permeabilized by 0.5% Triton X-100 in PBS for 2 min. Coverslips were washed with PBS-T and embedded with VectaShield with DAPI mounting media. Images were acquired with identical settings using a Zeiss 710 confocal microscope using “best signal” setting (Smart Setup, ZEN software, Zeiss).

### Plant materials and transgenic constructs

The transgenic lines FRI^+^ pVIN3:VIN3-eGFP/*vin3-4* (VIN3-GFP), FRI^+^ pVRN5:VRN5-YFP/*vrn5-8* (VRN5-YFP), FRI^+^ pFLC:FLC-WT/*flc-2* FRI, and FRI^+^ pFLC:FLC-C585T/*flc-2* FRI were described previously (Greb et al. 2007; Qüesta et al. 2016; Yang et al. 2017). The VIN3-GFP *vin3 vrn5* line was obtained by crossing pVIN3:VIN3-GFP *vin3-4* FRI^SF2^ into the *vrn5-8* FRI^SF2^ mutant (SALK_136506 crossed into Col*FRI*^SF2^). Primers used for PCR genotyping to identify homozygous plants are listed in Supplemental Table S1. The VEL1-3xFLAG construct was generated for this study by classic PCR cloning. The construct encoded *VEL1* genomic DNA from −2100 bp upstream of the ATG to 720 bp downstream from the stop codon. The 3xFLAG tag was inserted at the C terminus by PCR. The construct was cloned into the binary vector pCAMBIA1300 by restriction cloning and transformed into *vel1-1* FRI^SF2^/fri. After identification of plants that contained the transgene, plants were made homozygous for FRI^SF2^ to give rise to VEL1-3xFLAG/*vel1* FRI^SF2^. To generate the VRN2-Venus line, the genomic *VRN2* sequence from Col-0 was cloned from −1364 up upstream of the ATG and fused with C-terminal Venus excluding the native VRN2 stop codon. T3A was used as a terminator sequence. The construct was cloned into the binary vector pCAMBIA1300 by restriction cloning and transformed into *vrn2-1* Col*FRI* (Yang et al. 2017) mediated by agrobacterium C58 using the floral dip method.

### Co-IP assays in plants

Total proteins were extracted from 6 g of frozen ground *Arabidopsis* seedling tissue with IP buffer (50 mM Tris-HCl at pH 7.5, 150 mM NaCl, 0.5% NP-40, 1% Triton, EDTA-free protease inhibitor cocktail [Roche]). Lysates were cleared by centrifugation at 6000*g* for 30 min at 4°C and incubated with GFP-Trap (Chromotek) for 2 h. Immunoprecipitates were washed four times with IP buffer and eluted by boiling in 4× NuPAGE LDS sample buffer for 10 min. Input and co-IP fractions were separated by polyacrylamide gel electrophoresis (SDS-PAGE) and blotted onto polyvinylidine difluoride (PVDF) membranes. Primary antibodies anti-GFP (Roche 11814460001) and anti-FIE (Agrisera AS12 2616) were diluted 1:1000, and anti-Actin (Agrisera AS132640) was diluted 1:5000. Secondary antibodies were HRP-coupled. Blots were washed with TBS containing 0.05% Tween-20 and developed with SuperSignal West Femto maximum sensitivity substrate (Thermo Scientific).

### ChIP and ChIP-seq

For all ChIP analyses, plant material was cross-linked by vacuum-infiltrating seedlings in a solution of 1% (w/v) formaldehyde in PBS buffer for 10 min. The cross-linking reaction was stopped by adding glycine to a final concentration of 0.125 M followed by 5 min of vacuum infiltration. Nuclei were extracted from 2 g of ground frozen material with Honda buffer (0.44 M sucrose, 1.25% Ficoll, 2.5% Dextran T40, 20 mM HEPES KOH at pH 7.4, 10 mM MgCl_2_, 0.5% Triton X-100, 5 mM DTT, protease inhibitors) and washed with Honda buffer several times. For histone ChIP, chromatin was extracted with nucleus lysis buffer (50 mM Tris/HCl at pH 8, 10 mM EDTA, 1% [w/v] SDS, PIC) and sonicated using the Bioruptor standard (Diagenode) for 15 cycles (30 sec on/ 30 sec off) to achieve an average fragment size of 200–500 bp. After removing cellular debris by centrifugation at 10,000*g* for 10 min, the chromatin was diluted 10-fold with ChIP dilution buffer (1.1% [v/v] Triton X-100, 1.2 mM EDTA, 16.7 mM Tris/HCl at pH 8, 167 mM NaCl). An aliquot corresponding to 1% (v/v) of the starting chromatin volume was removed as the input DNA control. Immunoprecipitation was performed with Dynabeads protein A (Invitrogen) and α-H3K27me2 (Upstate Biotechnology 07-452) or α-H3 antibody (Abcam ab1791) for 4 h at 4°C on a rotator. Immunocomplexes were washed twice each with low-salt wash buffer (150 mM NaCl, 0.1% [w/v] SDS, 1% [v/v] Triton X-100, 2 mM EDTA, 20 mM Tris/HCl at pH 8), high-salt wash buffer (500 mM NaCl, 0.1% [w/v] SDS, 1% [v/v] Triton X-100, 2 mM EDTA, 20 mM Tris/HCl at pH 8), and TE buffer (1 mM EDTA, 10 mM Tris/HCl at pH 8) and eluted twice with freshly prepared elution buffer (1% [w/v] SDS, 0.1 M NaHCO_3_) by incubating in a ThermoMixer at 1000 rpm for 15 min at 65°C. NaCl was added to the eluates and the input DNA aliquots to a final concentration of 0.2 M; samples were then incubated at 600 rpm overnight at 65°C for decross-linking and treated with proteinase K for 1 h at 45°C. DNA was purified with phenol/chloroform extraction and eluted into water. Histone ChIP samples were tested for enrichment by qPCR with primer sequences for *FLC* and *ACTIN* (negative control) as previously published (Yang et al. 2017).

Nonhistone ChIP followed by qPCR analysis was performed as described above with the following modifications: 3 g of plant material was cross-linked for 15 min. After nuclei were resuspended in RIPA buffer (1× PBS, 1% Igepal CA-630, 0.5% sodium deoxycholate, 0.1% SDS, Roche Complete tablets) and the DNA was fragmented to 200–500 bp by sonication. anti-GFP (Abcam ab290) and Protein A agarose/salmon sperm DNA (Millipore 16–157) were used for IP of GFP-tagged and YFP-tagged proteins. For FLAG IP, anti-FLAG (Sigma F1804) was coupled to Dynabeads M-270 epoxy (Invitrogen 14301) following the manufacturer's protocol (2 µL of anti-FLAG coupled to 1.5 mg of epoxy beads was used for each IP reaction). VRN2 ChIP was performed with the following further modifications: After a brief sonication to rupture nuclei, chromatin was treated with benzonase (0.002 U/µL chromatin; Millipore 70746-4) for 15 min at 4°C to achieve a fragmentation of 200–500 bp. Benzonase was inactivated by adding EDTA to a final concentration of 10 mM before proceeding to IP. After IP, the EDTA concentration in all wash buffers was doubled.

Nonhistone ChIP-seq was performed as described above with the modifications that rProtein A sepharose Fast Flow (Merck GE17-1279-01) was used instead of Protein A agarose/salmon sperm DNA to avoid salmon sperm DNA contamination. Each biological replicated was generated by pooling three individual ChIP pull-downs (each consisting of 6 g of whole seedling tissues) during DNA purification using the ChIP DNA Clean & Concentrator kit (Zymo Research D5201). The samples were sent to BGI Genomics for library preparation and sequencing using DNBSEQ. At least two biological replicates were sequenced for each genotype and condition (*n* = 3 for VIN3-eGFP 6WT0/VRN5-YFP NV/VRN5-YFP 6WT0/VEL1-3xFLAG NV/VEL1-3xFLAG 6WT0, *n* = 2 for VIN3-eGFP NV/ColFRI-GFP IP NV/ ColFRI-GFP IP 6WT0/ ColFRI-FLAG IP NV, and *n* = 1 for ColFRI-FLAGIP 6WT0).

### ChIP-seq analysis

ChIP-seq reads were mapped to the TAIR 10 genome using the BWA (version 0.5.7) aligner with the parameters “-t 8 -l 25 -k 2 -n 5,” and the resulting SAM alignment records were converted to BAM using the SAMtools (version 1.6). The peaks were called from the BAM output using the call peak module from the MACS3 (version 3.0.0a7; https://github.com/macs3-project/MACS) with the following parameters: “-q 0.05 ‐‐bdg ‐‐nomodel ‐‐extsize 180.” The peak and the corresponding BAM output were visualized using the Integrative Genomics Viewer (IGV). Consensus peaks were called from peak regions that were present in at least two replicates. Consensus peaks were used to identify overlapping genes using bedtools (version 2.27.1). The peaks were further processed and compared using the bespoke Python scripts available at https://github.com/threadmapper/VEL-ChIPseq. A peak summit located within 1 kb upstream of the TSS to 1 kb downstream from the TTS was assigned to that gene. If multiple genes could be allocated to a peak, the closest gene was selected. If no gene was located within 1 kb of the peak, the peak was not assigned to any gene. For the metagene plot in Figure 7D, the replicates were merged. For comparison with CLF and SWN (Shu et al. 2019), the raw data were downloaded and reanalyzed with the same script used to analyze our VEL ChIP-seq.

### Data availability

The ChIP-seq raw data generated in this study has been deposited in the Sequence Read Archive (SRA) under project number PRJNA973989.

## Supplementary Material

Supplement 1

Supplement 2

Supplement 3
