# Supplementary material for: Distinct accessory roles of Arabidopsis VEL proteins in Polycomb silencing
Source: Genes Dev. Author manuscript; Available in PMC 2023 Oct 25. (PMC7615239; doi:10.1101/gad.350814.123)
Supplement: Supplementary Material [file EMS189790-supplement-Supplementary_Material.pdf]

Supplemental Material for:

Distinct accessory roles of Arabidopsis VEL proteins in Polycomb silencing.

Franco-Echevarría et al.

A

PLANTS	MAMMALS	DROSOPHILA
SWN	EZH1/2	E(z)
VRN2	SUZ12	Su(z)12
FIE	EED	Esc
MSI1	RBAP46/RBAP48	Nurf55

B

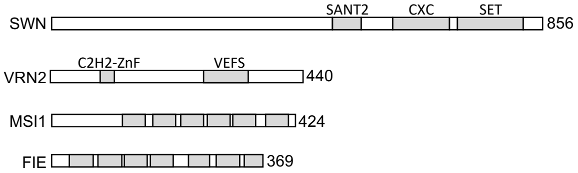

C

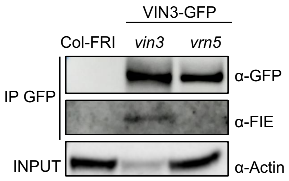

D

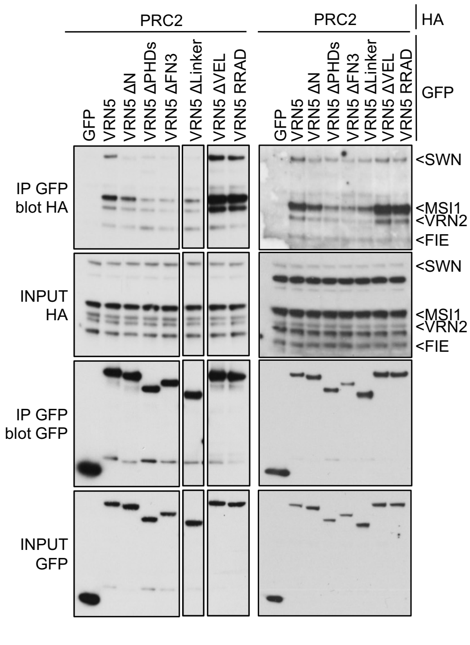

E

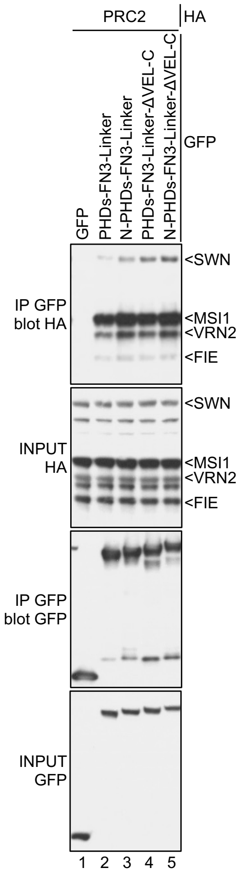

F

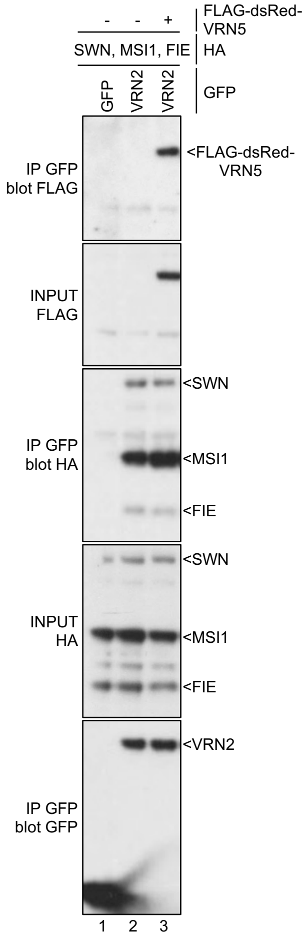

G

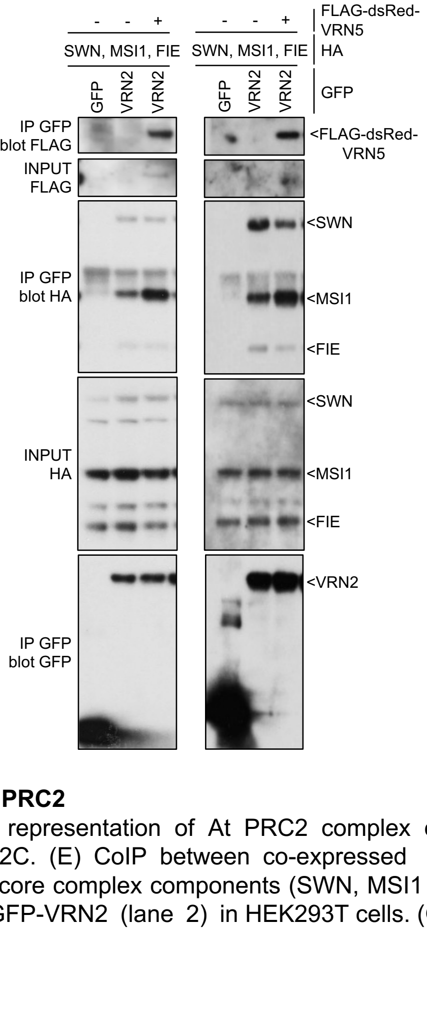

Supplemental Figure S1. Tight association between VRN5 and PRC2
(A) PRC2 core subunits in different organisms. (B) Schematic representation of At PRC2 complex core components. (C) Replicates of Fig. 2B. (D) Replicates of Fig. 2C. (E) CoIP between co-expressed proteins as indicated above panels. (F) CoIP of HA-tagged PRC2 core complex components (SWN, MSI1 and FIE) after co-expression with FLAG-dsRED-VRN5 (lane 3) or GFP-VRN2 (lane 2) in HEK293T cells. (G) Replicates of Fig. S1F.

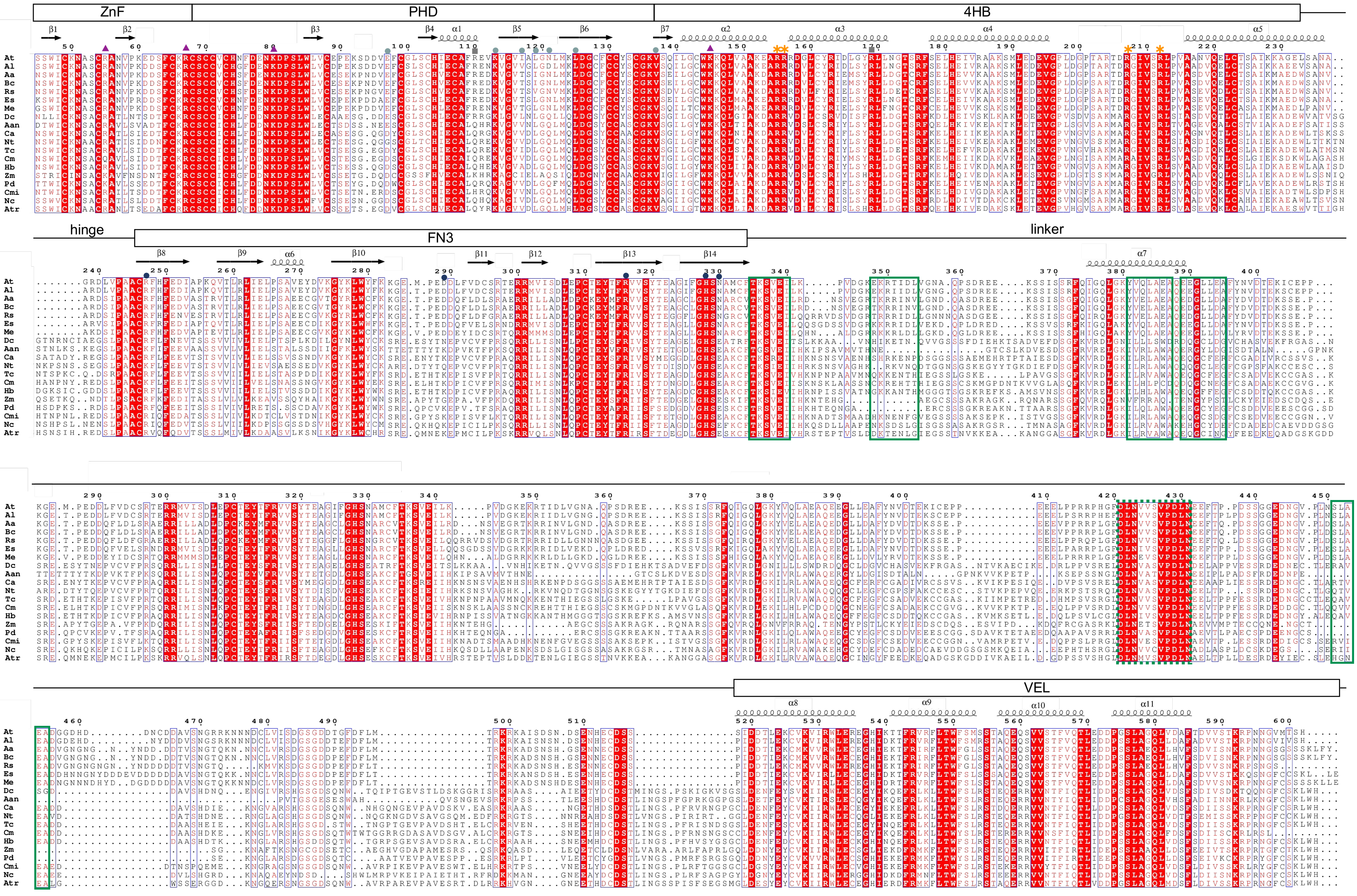
ZnF
PHD
4HB
hinge
FN3
linker
VEL

Supplemental Figure S2. Sequence conservation amongst VRN5 orthologs

Sequence alignments of VRN5 PHDsuper-FN3-linker-VEL segments from diverse angiosperm species (At, *Arabidopsis thaliana*; Al, *Arabidopsis lyrata*; Aa, *Arabis alpina*; Bc, *Brassica carinata*; Rs, *Raphanus sativus*; Es, *Eutrema salsugineum*; Me, *Microthlaspi erraticum*; Dc, *Daucus carota;* Aan*, Artemisa annua; Ca, Coffeea arabica;* Nt*, Nicotiana tabacum;* Tc, *Theobroma cacao*; Cm, *Cucumis melo*; Hb, *Hevea brasiliensis*; Zm, *Zostera marina*; Pd, *Phoenix dactylifera*; Cmi, *Cinnamomum micranthum;* Nc, *Nymphaea colorata*; Atr, *Amborella trichopoda*; see also Fig. S4); white in red boxes, invariant residues; red in blue frames, similar residues across species; predicted secondary structure elements indicated above; light blue and teal dots, PHDsuper and FN3 residues mediating inter-domain contacts within VRN5; green squares, conserved motifs mediating VRN5-MSI1 interaction (http://elm.eu.org/); dashed green square, VRN5-defining DLNxxxVPDLN motif; purple triangles, patch 1 residues (R55, R67, K80 and K146); orange asterisks, patch 2 residues (R155, R156, R207 and R212); grey square, patch 3 residues (R110 and R169).

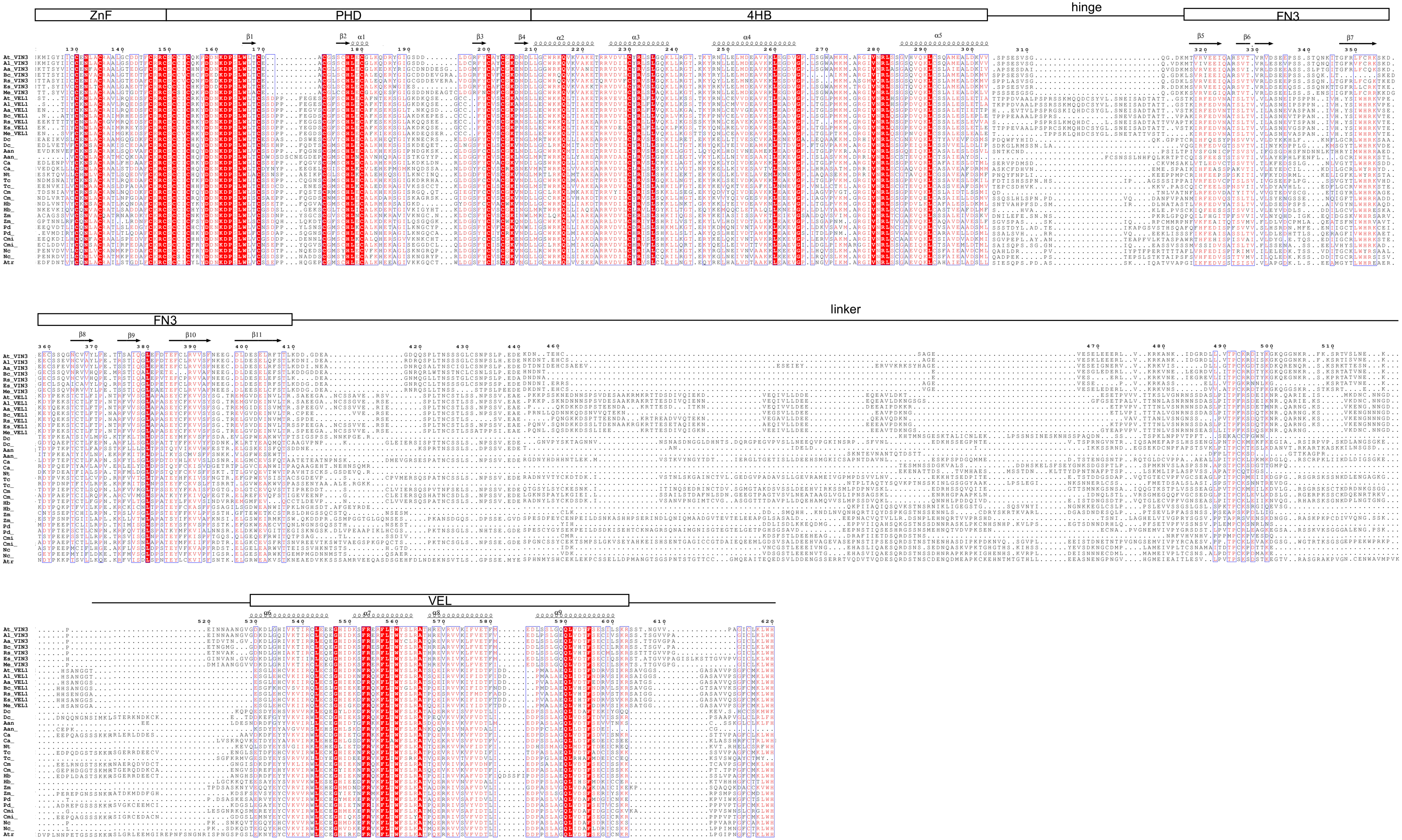

ZnF
PHD
4HB
hinge
FN3
FN3
linker
VEL

Supplemental Figure S3. Sequence conservation amongst VIN3/VEL1 orthologs

Sequence alignments of VIN3/VEL1 PHDsuper-FN3-linker-VEL segments from diverse angiosperm species as described in Fig. S2; white in red boxes, invariant residues; red in blue frames, similar residues across species; predicted secondary structure indicated above.

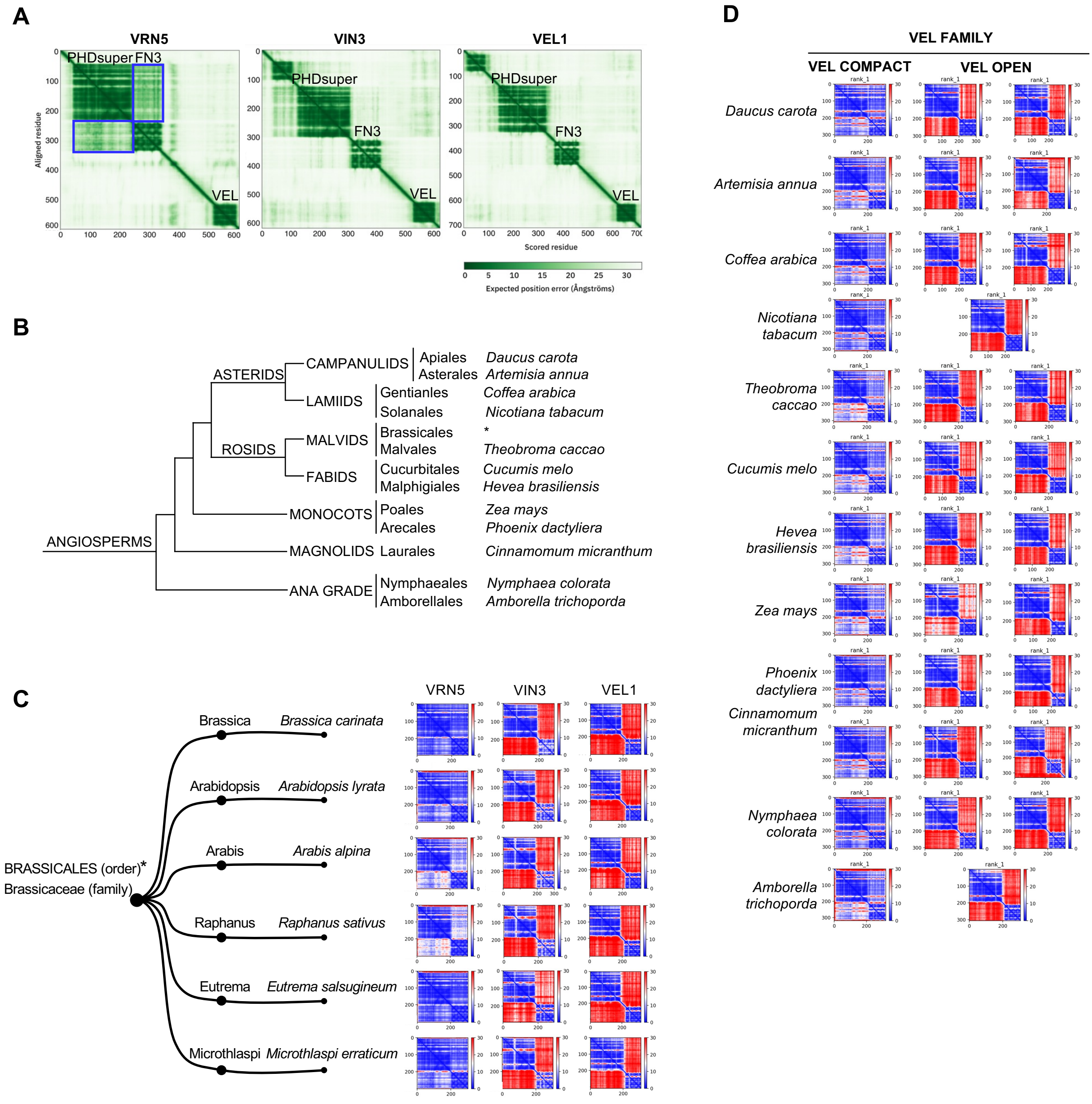

Supplemental Figure S4. VRN5-specific inter-domain contacts between PHDsuper and FN3 domains
(A) PAE plot for full-length VRN5, VIN3 and VEL1 proteins calculated by AlphaFold2, predicting the relative positions between domains. Shades of green indicate expected distance error (in Å) between pairs of residues; dark green, low error; light green, high error. Note that the PAE plot for VRN5 albeit not for VIN3 or VEL1 suggests tight packing between PHDsuper and FN3 domains, marked with a blue square. The backbones of the predicted PHDsuper-FN3 structures of VRN5 orthologs exhibit low RMSD and high template-modeling (TM) score values, ranging from 0.81 to 0.94, whereby 1 indicates a perfect match between two structures. (B) Phylogenetic tree of angiosperm species included in this study. (C) Phylogeny of *Brassicales* species included in this study, with PAE plot for VRN5, VIN3 and VEL1 PHDsuper and FN3 domains; blue, low error; red, high error. (D) PAE plots for VRN5, VIN3 and VEL1 PHDsuper and FN3 domains from different angiosperms as in Fig. S4B.

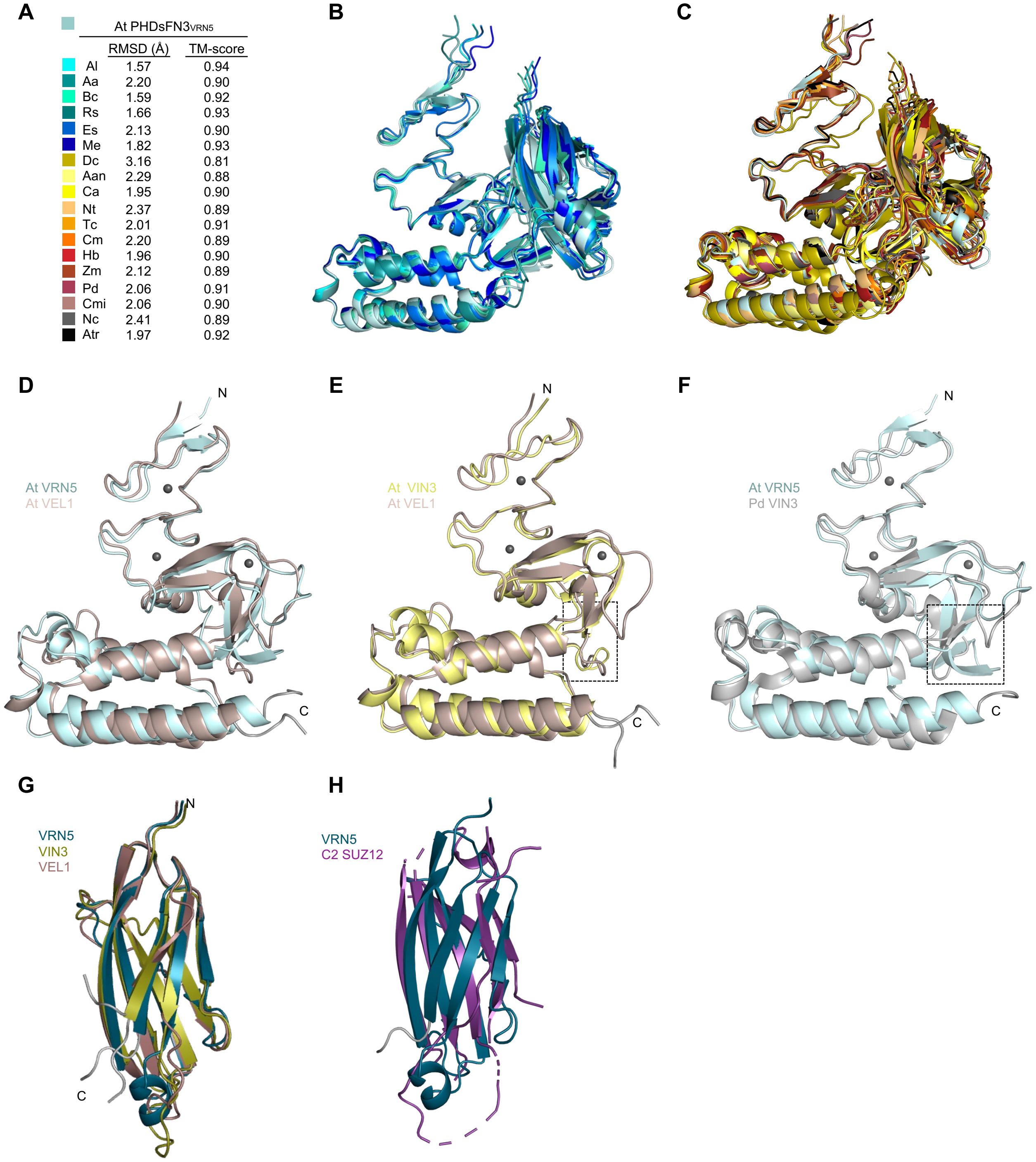

Supplemental Figure S5. Compact fold of VRN5 mediated by conserved PHDsuper-FN3 contacts

(A) RMSD and TM-score values for predicted VRN5 PHDsuper-FN3 structures from various angiosperm species (as in Fig. S4B) relative to the predicted At VRN5 PHDsuper-FN3 structure. (B) Superposition of structure predictions for VRN5 PHDsuper-FN3 from different Brassicaceae species; and (C) from different angiosperms (see Figs. S4B, C and D), with color code as in A. (D-H) Superpositions of various domains in ribbon representation, predicted by AlphaFold2, with N- and C-termini indicated; (D) PHD superdomains of At VRN5$_{41-240}$ (light blue) versus At VEL1$_{143-334}$ (light brown); grey balls, zinc ions; RMSD 2.30 Å. (E) PHD superdomains of At VIN3$_{123-307}$ (light yellow) versus At VEL1$_{143-334}$ (light brown); RMSD 1.79 Å; dashed square, missing bulky beta region. (F) PHD superdomains of At VRN5$_{41-240}$ (light blue) versus Pd VIN3 (light grey, PDB 7QCE); RMSD 2.33 Å. (G) FN3 domains of At VRN5$_{242-339}$ (teal), VIN3$_{313-412}$ (gold) versus VEL1$_{366-462}$ (brown); gray, hinge residues; FN3$_{VRN5}$ versus FN3$_{VEL1}$, RMSD 2.06 Å; FN3$_{VEL1}$ versus FN3$_{VIN3}$, RMSD 2.46 Å, and FN3$_{VRN5}$ versus FN3$_{VIN3}$, RMSD 2.28 Å. (H) FN3 domains of At FN3$_{VRN5}$ (teal) versus mammalian C2 domain from SUZ12 (purple), RMSD 3.94 Å.

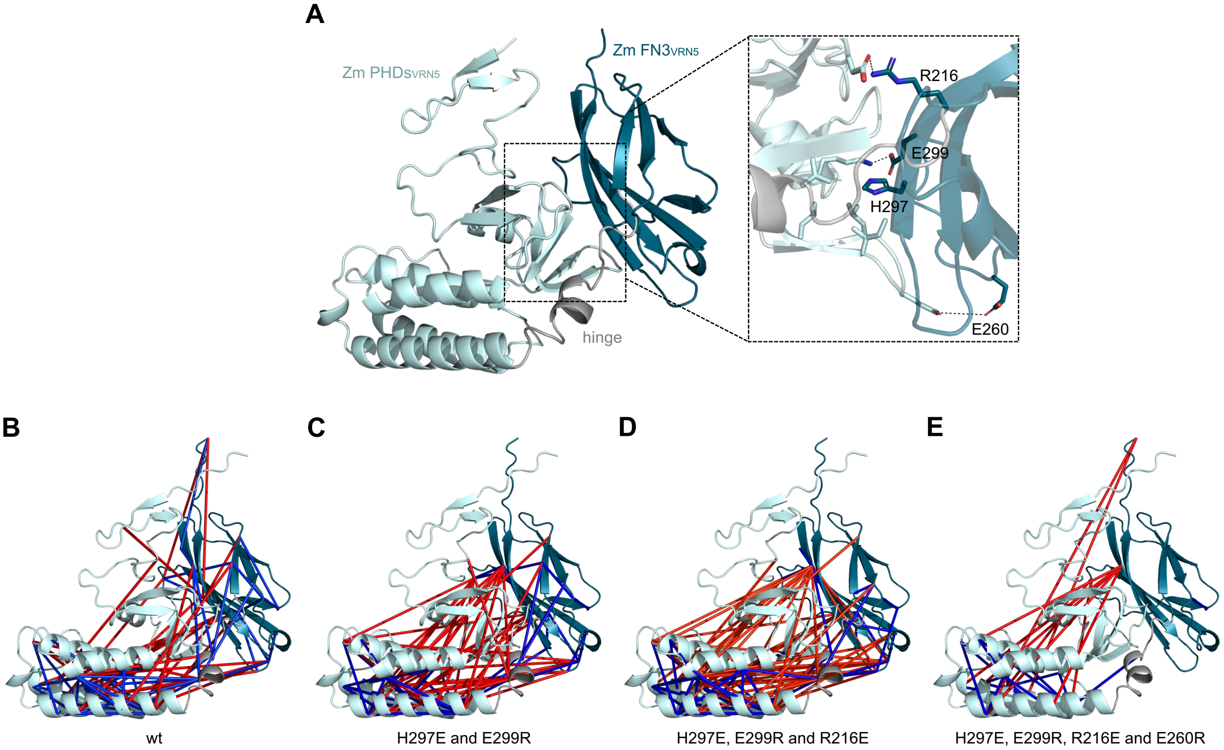

Supplemental Figure S6. Analysis of VRN5 PHDsuper-FN3 interface by XL-MS

(A) Structure prediction for *Zea mays* (Zm) PHDsuper-FN3$_{VRN5}$ by AlphaFold2; light blue, PHDsuper domain; teal, FN3 domain. Close-up view of the interactions between Zm PHDsuper and FN3, with mutated residues mediating interactions shown in stick representation; dashed lines, hydrogen bond and salt bridges. (B-E) Cross-links in wt and mutants Zm PHDsuper-FN3$_{VRN5}$ mapped onto structures predicted by AlphaFold2; cross-link above (red) or below (blue) the limit of 27 Å are indicated. Repulsive point mutations in key residues mediating these cross-links (H297E and E299R) blocked several of these cross-links, leading to an increased degree of freedom between the constituent domains. This is even more pronounced if a third repulsive mutation is introduced, which caused large (>27Å) structural alterations. Finally, if four predicted interface residues were mutated (H297E, E299R, R216E, E260R), most of the inter-domain cross-links disappeared, whereby the only remaining ones were within the 4HB module of PHDsuper.

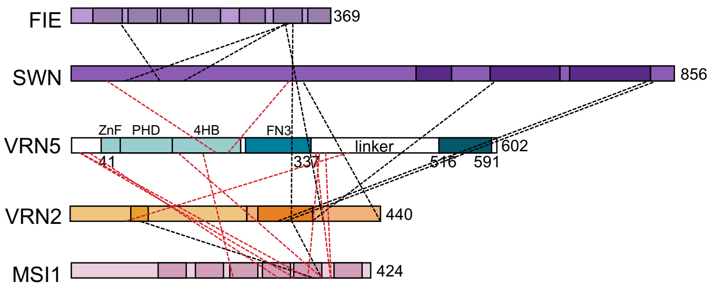

Supplemental Figure S7. Schematic representation of XL-MS results for VRN5 ΔVEL-PRC2 complex
Domain organization of VRN5 and PRC2 as described in Fig. 1A, Fig. 5A & Supplemental Fig. S1.
Intra PRC2 complex cross-links found by XL-MS in black, and inter VRN5-PRC2 cross-links identified in red.

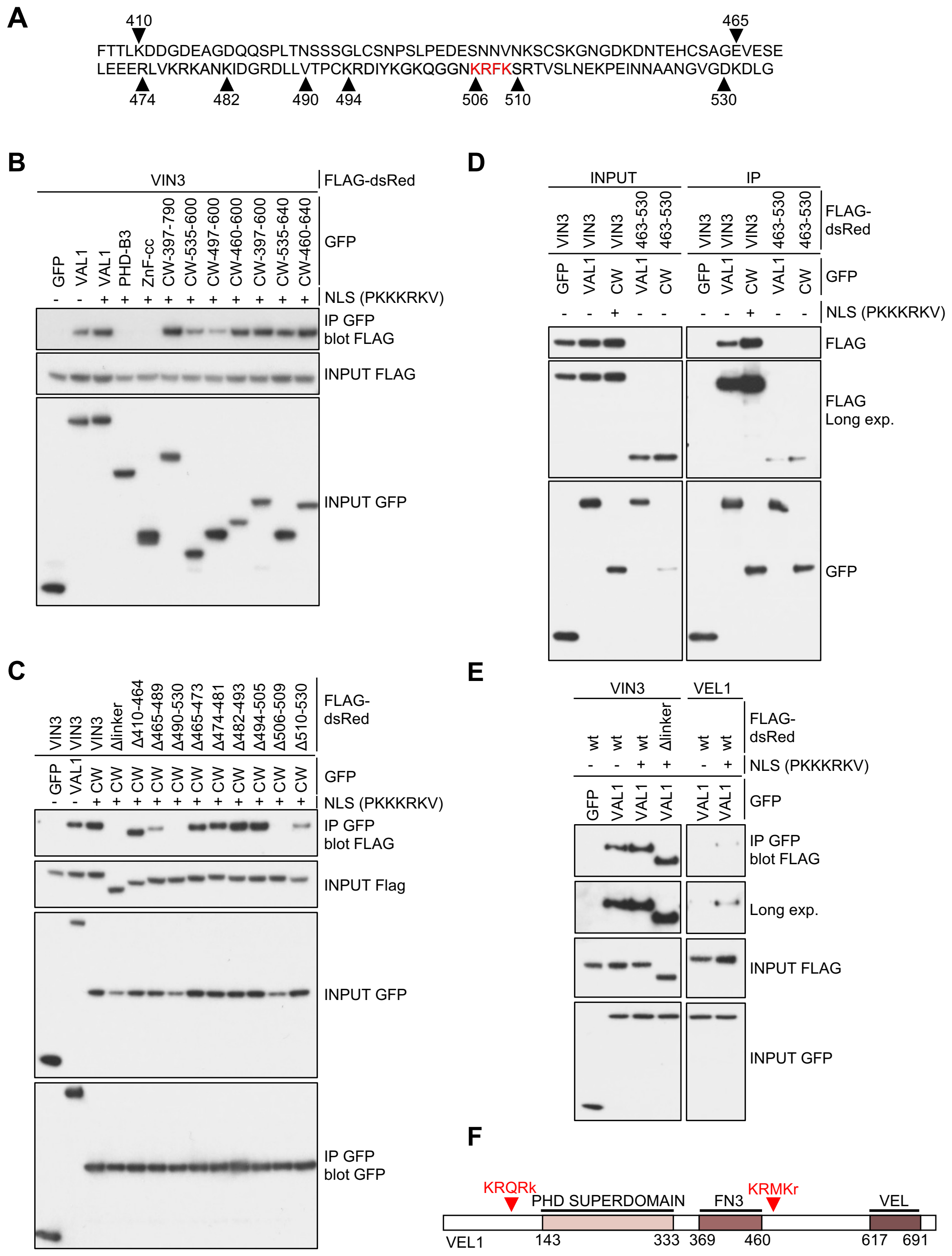

Supplemental Figure S8. Association of VIN3 with VAL1

(A) Flexible linker sequence separating At VIN3 FN3 and VEL domains (410-530), with linker residues indicated by arrowheads; *red*, KRFK motif. (B) CoIP assays between VAL1 and VIN3 domains as indicated in panels bearing a viral NLS (PKKKRKV) next to the N-terminal tag with wt VIN3. (C) CoIP assays of $CW_{460\text{-}640}$ with wt and mutant VIN3, as indicated, revealing the critical role of KRFK for the association between VIN3 and VAL1 (same results as with full-length GFP-VAL1). (D) CoIP assays of VAL1 or $CW_{460\text{-}640}$ (+/- PKKKRKV) with wt VIN3 or a minimal linker region of $VIN3_{463\text{-}530}$, revealing that a short KRFK-containing linker fragment of VIN3 is sufficient to mediate weak association with $CW_{460\text{-}640}$. (E) CoIP assays of VAL1 +/- a viral NLS (PKKKRKV) next to its N-terminal tag, and wt or deletion mutants of VIN3, revealing that the association between VAL1 and VIN3 deletions lacking KRFK can be restored to some extent by a heterologous viral NLS (as its sequence is highly similar to KRFK). VEL1-VAL1 interaction is also increased by the insertion of viral NLS in VEL1. (F) Domain architecture of At VEL1; *red*, putative monopartite NLS motifs.

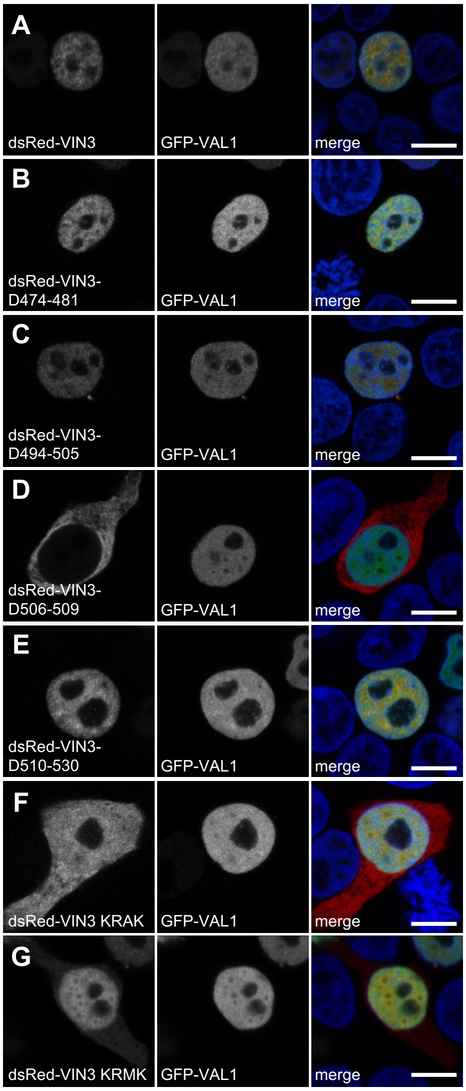

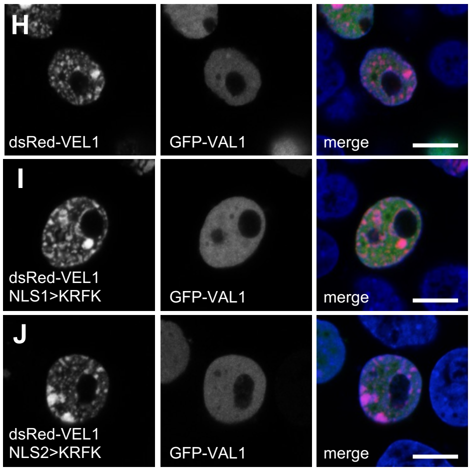

K

**Mutants**	**Cellular localization**
NLS-dsRed-VIN3	Nuclear
dsRed-VIN3 (aa463-530)	Nuclear
dsRed-VIN3 Δlinker(410-530)	Cytoplasmic
dsRed-VIN3 Δ410-464	Nuclear
dsRed-VIN3 Δ465-489	Nuclear-Cytoplasmic
dsRed-VIN3 Δ490-530	Cytoplasmic
dsRed-VIN3 Δ465-473	Nuclear
dsRed-VIN3 Δ482-493	Nuclear
GFP-NLS-VAL1	Nuclear
GFP-NLS-CW(460-640)	Nuclear
NLS-dsRed-VEL1	Nuclear

Supplemental Figure S9. Subcellular distribution of VIN3 and VAL1 mutants
(A-J) Representative confocal images of HEK293T cells transfected with wt GFP-VAL1 (green in merges) and wt or mutant dsRed-VIN3 (red in merges), after fixation and staining with DAPI (blue in merges) to mark nuclei, as indicated in panels; scale bars 10 μm. (K) Summary of subcellular localizations of wt or mutant VIN3 tested for VAL1 association by coIP following co-expression with VAL1 (see also Figs. 6 & S8).

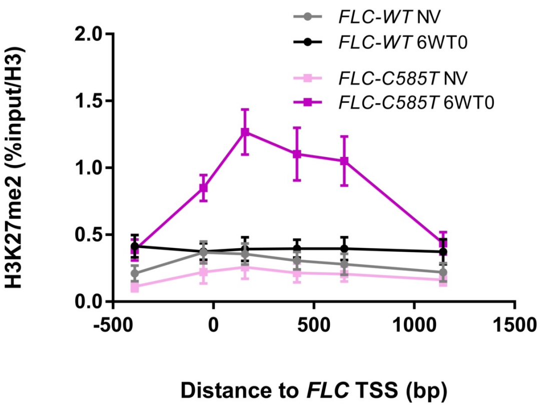

Supplemental Figure S10. A mutation preventing binding of VAL1 at *FLC* results in the accumulation of H3K27me2 in the nucleation region in the cold. Enrichment of H3K27me2 in plants carrying a *FLC* transgene with a mutation in the first RY site (VAL1 binding motif) in *FLC* intron 1 (*FLC-C585T*). Transgenic *FLC-WT* plants were used as a control sample. Note that the 3' end of *FLC* was omitted from the analysis because this region of endogenous *FLC* is still present in the *flc-2* FRI mutant background of the transgenic plants. Data are shown as the percentage input relative to H3 for non-vernalized (NV) or vernalized plants (6WT0). Error bars are means ± s.e.m. from two independent experiments.

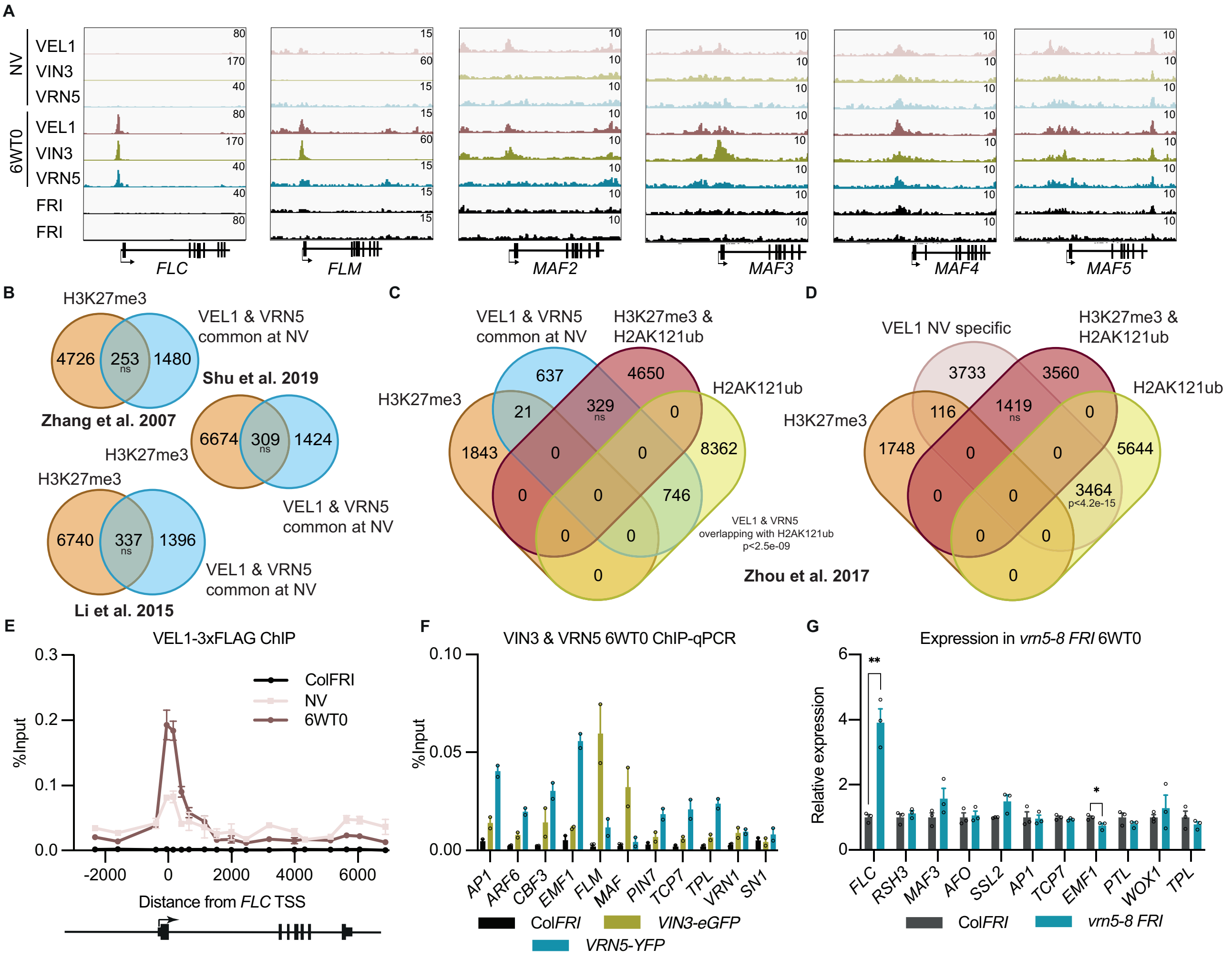

Supplemental Figure S11. Co-localization of VEL proteins with H3K27me3 and H2AK121ub
(A) IGV screenshots showing the enrichment of the VEL proteins (VEL1, VIN3, and VRN5) at non-vernalized (NV) and after six weeks of vernalization (6WT0) at the *FLC* clade genes. (B) Venn diagram showing the overlap between H3K27me3 marked genes from different studies (Zhang et al. 2007, Li et al. 2015, Shu et al. 2019) and potential target genes that are common between VEL1 and VRN5 at non-vernalized (NV) conditions. P values for overlaps were determined by hypergeometric tests. (C-D) Venn diagram showing the overlaps between genes marked by H3K27me3, H2AK121ub, and by both modification (Zhou et al. 2017) and potential target genes that are common between VEL1 and VRN5 (C) or that are specific for VEL1 (D) at NV conditions. P values for overlaps were determined by hypergeometric tests. (E) VEL1 ChIP-qPCR enrichment at *FLC* at NV and 6WT0. Data are shown as the percentage input. Non-transgenic Col*FRI* plants were used as a negative control sample. Error bars are means ± s.e.m. from two independent experiments. The gene model of *FLC* is shown underneath the graph. (E) ChIP-qPCR showing enrichment of VIN3 and VRN5 at ten potential target genes at 6WT0. Data are shown as the percentage input. Non-transgenic Col*FRI* plants were used as a negative control sample, and *SN1* was used as a negative control locus. Error bars are means ± s.e.m. from two independent experiments. (F) Expression of several potential target genes in the mutant *vrn5-8 FRI* relative to wt Col*FRI* at 6WT0. Data are shown normalized to the expression level of the respective gene in Col*FRI*. Error bars are means ± s.e.m. from at least three biological replicates. For statistical tests, a single asterisk denotes p<0.05, and two asterisks denote p<0.01 between samples by Student's t-test.

Table S1. Primers used for genotyping

Primer name	Sequence
vin3-4 WT allele_F	CCAGAGAACATGGATTCTTCTTCG
vin3-4 WT allele_R	CTAAGCATCAGAAAGAAATTGG
vin3-4 T-DNA_R	CTAAATTCTCACAGCAAATGATGTAA
vin3-4 T-DNA border (vin3-4 T-DNA_R)	CATTTTATAATAACGCTGCGGACATCTAC
vrn5-8 RP	TCACATTGAGTGTGCTTTTCG
vrn5-8 LP	CTCCTCATTCAAGTCTGGCAC
LbB1.3, with vrn5-8_RP	ATTTTGCCGATTTCGGAAC
